# Exendin-4 promotes retinal ganglion cell survival and function by inhibiting calcium channels in experimental diabetes

**DOI:** 10.1016/j.isci.2023.107680

**Published:** 2023-08-18

**Authors:** Yong-Chen Wang, Lu Wang, Yu-Qi Shao, Shi-Jun Weng, Xiong-Li Yang, Yong-Mei Zhong

**Affiliations:** 1 State Key Laboratory of Medical Neurobiology and MOE Frontiers Center for Brain Science, Institutes of Brain Science, Fudan University, 138 Yixueyuan Road, Shanghai 200032, China

**Keywords:** Endocrinology, Molecular biology, Endocrine regulation

## Abstract

Progressive damage of retinal ganglion cells (RGCs) is observed in early diabetic retinopathy. Intracellular Ca^2+^ overload mediated by Ca^2+^ influx through voltage-gated Ca^2+^ channels (VGCCs) is involved in neurodegeneration, whereas glucagon-like peptide-1 (GLP-1) provides neuroprotection. However, whether GLP-1 plays a neuroprotective role in diabetic retinas by modulating VGCCs remains unknown. We found that eye drops of exendin-4, a long-acting GLP-1 receptor (GLP-1R) agonist, prevented the increase of L-type Ca^2+^ current (*I*_LCa_) densities of RGCs induced by 4-week hyperglycemia and promoted RGC survival by suppressing L-type VGCC (L-VGCC) activity in streptozotocin-induced diabetic rats. Moreover, exendin-4-induced suppression of *I*_LCa_ in RGCs may be mediated by a GLP-1R/Gs/cAMP-PKA/ryanodine/Ca^2+^/calmodulin/calcineurin/PP1 signaling pathway. Furthermore, exendin-4 functionally improved the light-evoked spiking ability of diabetic RGCs. These results suggest that GLP-1R activation enhances cAMP to PP1 signaling and that PP1 inactivates L-VGCCs by dephosphorylating them, thereby reducing Ca^2+^ influx, which could protect RGCs against excitotoxic Ca^2+^ overload.

## Highlights

• Four-week hyperglycemia increases L-type Ca^2+^ current (*I*_LCa_) density of rat RGCs
• Eye drops of the GLP-1R agonist Ex-4 prevents diabetes-induced increase of *I*_LCa_
• Ex-4 promotes RGC survival by inhibiting L-type voltage-gated Ca^2+^ channels
• A Gs/cAMP-PKA/ryanodine/Ca^2+^/CaM/calcineurin/PP1 pathway mediates the Ex-4 effect

## Graphical abstract

**Figure:**
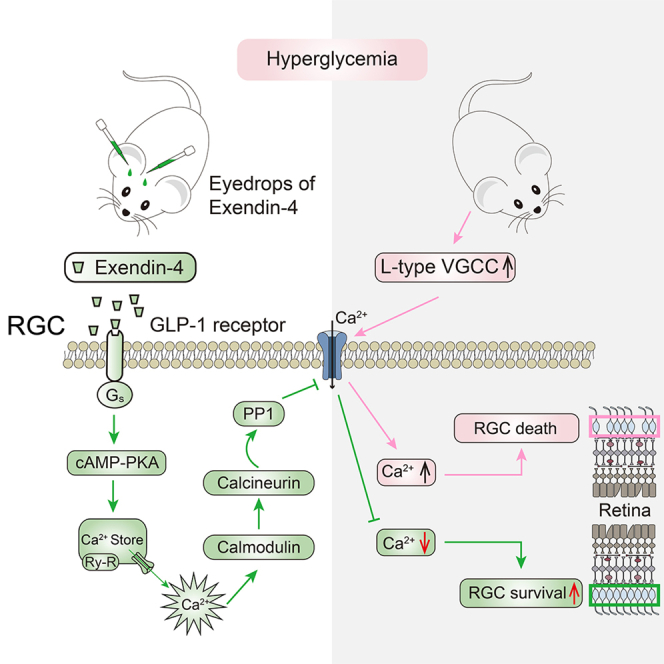

## Introduction

Diabetic retinopathy (DR) is a major complication of diabetes mellitus (DM) and remains the leading cause of blindness globally.^1^^,^^2^ Due to the complexity of the disease and researchers’ limited understanding of its pathogenic mechanisms, there are no effective therapeutic strategies for this disease. Increasing evidence indicates that neurodegeneration is an early event in the pathogenesis of DR that is linked to visual dysfunction or blindness.^3^^,^^4^^,^^5^^,^^6^^,^^7^^,^^8^^,^^9^ Retinal ganglion cells (RGCs), the only output neurons in the retina, are progressively damaged and lost in both patients and animal models with DM.^4^^,^^5^^,^^6^^,^^10^^,^^11^^,^^12^ Other researchers and we have previously observed remodeled RGC dendritic branching patterns and altered RGC membrane properties in several diabetic animal models.^4^^,^^11^^,^^13^^,^^14^^,^^15^^,^^16^

Ca^2+^ overload is caused by a massive Ca^2+^ influx through voltage-gated Ca^2+^ channels (VGCCs) and ionotropic glutamate receptor channels. It is harmful to the cells because it constitutes the final common pathway for neuronal death under various pathological conditions,^17^^,^^18^^,^^19^ including glaucoma patients and animal models, and rat models with retinal ischemia.^20^^,^^21^^,^^22^^,^^23^ However, no studies have investigated the changes and roles of VGCCs in RGCs for DR development. L-type voltage-gated Ca^2+^ channels (L-VGCCs) have been shown to be expressed in rat RGCs.^24^ L-VGCCs are high-voltage-activated ones that do not inactivate with time^25^; therefore, their continuous activation may contribute significantly to the lethal Ca^2+^ influx under pathological conditions.^22^ Based on these research backgrounds, the first purpose of this work was to explore whether L-VGCCs in RGCs are altered in streptozotocin (STZ)-induced diabetic rats, a model of human type 1-like diabetes.

Therapeutic strategies based on neuroprotection in the early stages of DR could be effective in preventing visual impairment. Glucagon-like peptide-1 (GLP-1) is a metabolic hormone secreted from intestinal endocrine L-cells that stimulates glucose-dependent insulin secretion.^26^ Activation of GLP-1 receptor (GLP-1R) provides neuroprotection in a variety of experimental models of neurodegenerative disorders.^27^^,^^28^ In addition, GLP-1 is expressed in the vertebrate retinas.^29^^,^^30^^,^^31^ Specifically, GLP-1R immunoreactivity has been detected in the ganglion cell layer (GCL) in human, rat and mouse retinas.^29^^,^^30^^,^^32^^,^^33^^,^^34^^,^^35^^,^^36^ The presence of GLP-1R in the retina could serve as a potential new target for treating neurodegeneration through GLP-1R agonists. Actually, GLP-1 and GLP-1R agonists have been shown to prevent electroretinogram abnormalities and retinal neurodegeneration, protect the blood retinal barrier and also affect autophagy in diabetic animal models.^12^^,^^29^^,^^32^^,^^33^ However, whether GLP-1 and GLP-1R agonists exert neuroprotective effects by affecting the cellular calcium system in the retina is still unknown, although Gilman et al. reported that GLP-1 suppressed Ca^2+^ currents and glutamate-induced currents in cultured rat hippocampal neurons.^37^

It has been shown that GLP-1 and longer-lasting protease-resistant GLP-1R agonists (Exendin-4, Val8GLP-1, liraglutide) can cross the blood-brain barrier to exert effective effects in the central nervous system,^38^^,^^39^^,^^40^^,^^41^^,^^42^^,^^43^ but whether they can cross the blood-retinal barrier remains unknown. If they do, because GLP-1R agonists have hypoglycemic effects, if administered systemically, it is difficult to determine whether the observed effects on neurodegeneration prevention are due to a decrease in blood glucose level or to the effect induced by direct activation of GLP-1R in the retina. Topical application of GLP-1R agonists with eye drops could help answer this question because this method seems unlikely to lower blood glucose levels and provides a noninvasive and effective means of administration. On this basis, the second question we wanted to address was whether and how topical administration of the GLP-1R agonist exendin-4 (Ex-4) plays a neuroprotective role in diabetic RGCs by modulating L-VGCCs in RGCs.

## Results

### DM reduces GLP-1 mRNA levels in the retina

**Figure 1:**
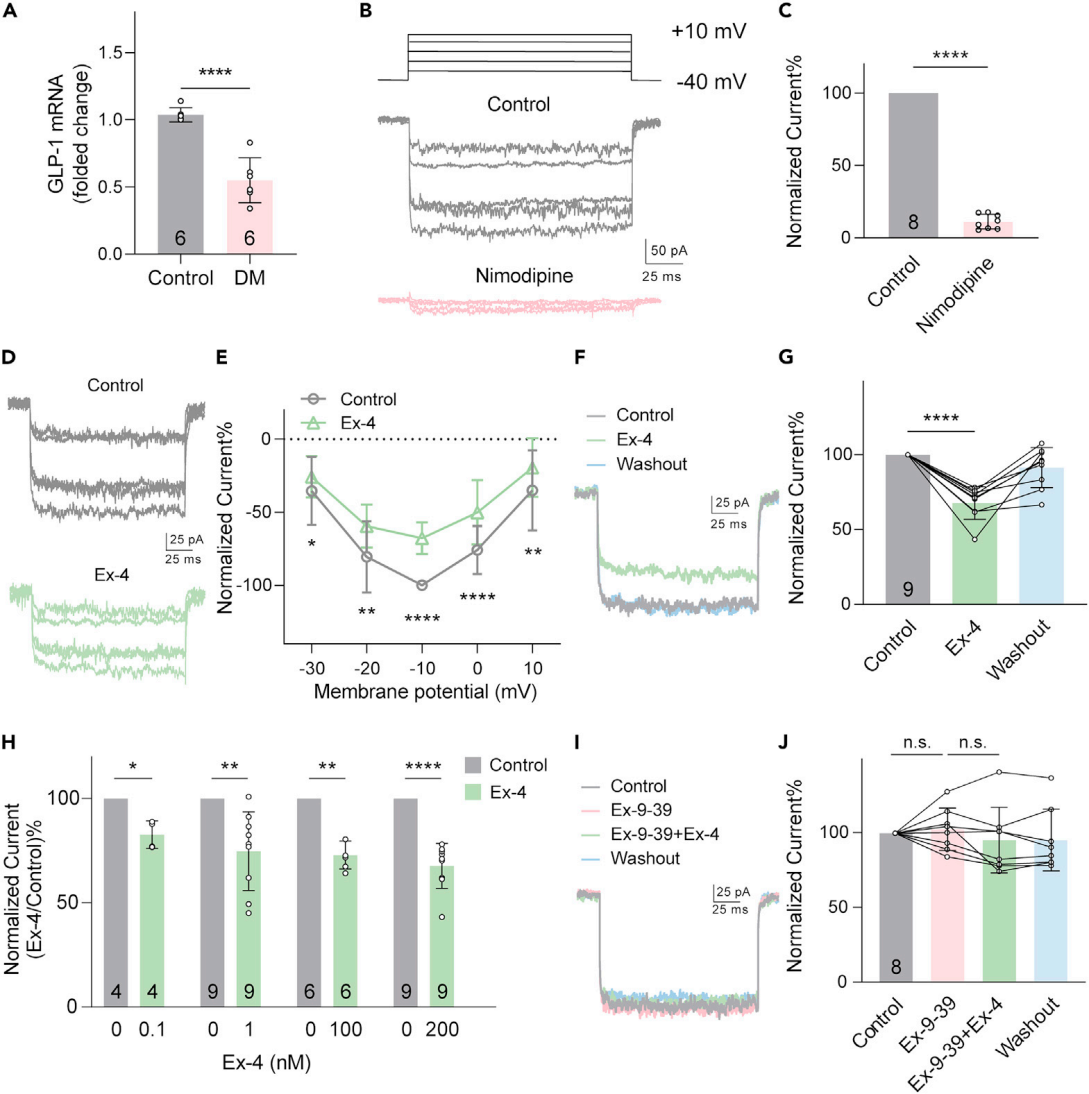
Ex-4 suppressed *I*_LCa_ in RGCs via GLP-1R (A) Real-time quantitative RT-PCR analysis showing a significant decrease in GLP-1 mRNA levels in DM retinas (n = 6, ∗∗∗∗p < 0.0001 by unpaired *t* test). Beta-2-microglobulin was used as a house-keeping gene. (B) Representative current recordings from an acutely isolated RGC. The cell was held at −40 mV, and the high-voltage activated Ca^2+^ currents were induced by depolarization voltage pulses from −30 mV to +10 mV in increments of 10 mV. The currents were almost completely suppressed by 10 μM nimodipine. (C) Bar chart summarizing the changes of current amplitudes after nimodipine application (n = 8). (D) Representative *I*_LCa_ traces of an RGC, showing that extracellular application of Ex-4 (200 nM) suppressed the current amplitudes. (E) Current–voltage (I–V) relationship curves showing that Ex-4 voltage-dependently suppressed the *I*_LCa_ amplitudes (n = 9). (F and G) Current traces and bar chart showing the effect of Ex-4 on *I*_LCa_ recorded at −10 mV (n = 9). (H) Ex-4 suppressed *I*_LCa_ in a dose-dependent manner. Only one concentration of Ex-4 was tested for a single cell. Each cell was recorded before Ex-4 perfusion (control) and during Ex-4 perfusion, and each Ex-4 response was then normalized against its corresponding control response, which is always 100%. Cell numbers are marked inside the bars. (I) Representative currents showing that 100 nM Ex-9-39 blocked the Ex-4 induced suppression of *I*_LCa_. (J) Bar chart summarizing the effects of Ex-9-39 and Ex-4 on *I*_LCa_ obtained at −10 mV (n = 8). Data are presented as mean ± SD. n.s., p > 0.05, ∗p < 0.05, ∗∗p < 0.01 and ∗∗∗∗p < 0.0001 by paired *t* test.

Assessment of GLP-1 expression by qRT-PCR showed a 1.8-fold reduction of specific mRNA levels in the retinas of STZ-induced DM rats after 4 weeks of hyperglycemia in comparison with age-matched control rats (p < 0.0001, Figure 1A).

### Ex-4 suppresses *I*_LCa_ in RGCs via GLP-1R

We next investigated the effect of Ex-4, a protease-resistant long-acting agonist of GLP-1R, on *I*_LCa_ from RGCs since the endogenous GLP-1 is highly sensitive to degradation by dipeptidyl peptidase Ⅳ.^44^ High-voltage-activated Ca^2+^ currents from an acutely isolated RGC were induced by a series of depolarizing pulses from −40 mV up to +10 mV in increments of 10 mV (Figure 1B). The currents were almost completely suppressed by perfusions of 10 μM nimodipine, a specific L-VGCC blocker, with the average current amplitudes at −10 mV being reduced to 11.19 ± 5.09% of control (p < 0.0001, Figures 1B and 1C). This suggests that the currents are mainly mediated by L-VGCCs.

Perfusion of 200 nM Ex-4 reduced *I*_LCa_ amplitudes (Figure 1D). The stable *I*_LCa_ amplitudes measured in nine RGCs are plotted as a function of membrane potential (I–V curve), showing that Ex-4 voltage-dependently suppressed the *I*_LCa_ (Figure 1E). For example, at −10 mV *I*_LCa_ was reduced to 67.70 ± 10.85% of control (p < 0.0001, Figures 1F and 1G). This Ex-4 effect was dose-dependent. As shown in Figure 1H, the currents were suppressed to 82.62 ± 6.62% (p < 0.05), 74.73 ± 18.91% (p < 0.01), 72.87 ± 6.75% (p < 0.01) and 67.70 ± 10.85% (p < 0.0001) of the control following Ex-4 incubation at concentrations of 0.1 nM, 1 nM, 100 nM and 200 nM, respectively. Based on these data, 200 nM Ex-4 was chosen for all the experiments to be subsequently described. Perfusion of 100 nM Ex-9-39, a competitive GLP-1R antagonist, had no effect on the *I*_LCa_ (102.60 ± 14.12% of control, p > 0.05 vs. control), and further co-application of Ex-4 did not change the currents (95.23 ± 21.89% of control, p > 0.05 vs. Ex-9-39; Figures 1I and 1J), suggesting the involvement of GLP-1R in the Ex-4 effect.

### DM upregulates *I*_LCa_ densities and these effects were improved by Ex-4

**Figure 2:**
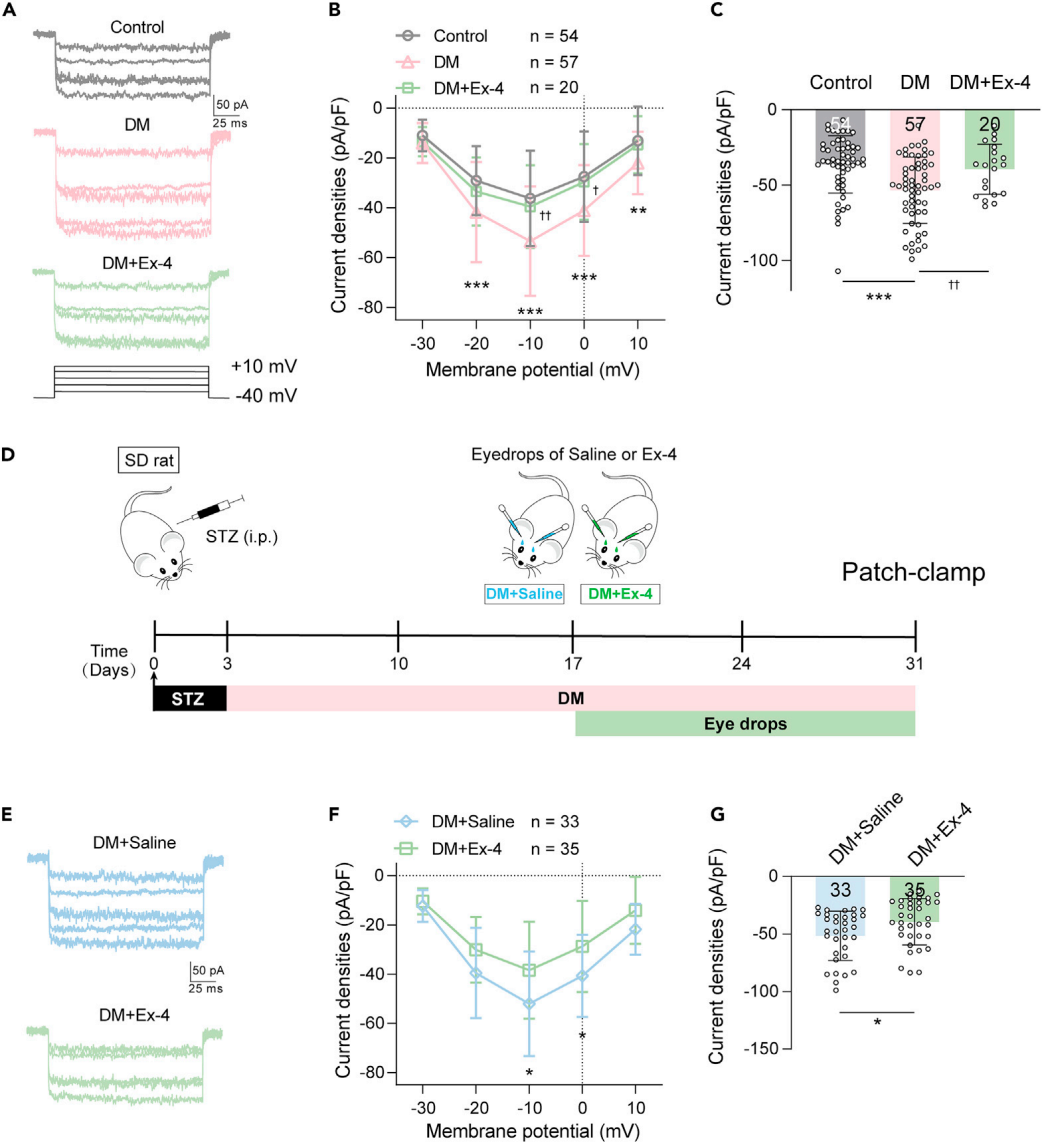
Ex-4 inhibited hyperglycemia-induced increases in *I*_LCa_ densities of RGCs (A) Representative *I*_LCa_ traces, recorded in isolated RGCs with similar membrane capacitance from control, and diabetic retinas before (DM) and after perfusion of Ex-4 (DM + Ex-4). The holding potentials of RGCs were set at −40 mV, and the currents were evoked in the range of −30 mV to +10 mV in increments of 10 mV. (B) Comparison of the I–V relationship curves of *I*_LCa_ in the RGCs of the control (n = 54), DM (n = 57) and DM + Ex-4 retinas (n = 20) (∗∗p < 0.01 and ∗∗∗p < 0.001 for DM vs. control; †p < 0.05 and ††p < 0.01 for DM vs. DM + Ex-4 by Sidak’s multiple comparisons test after two-way RM ANOVA). (C) Bar chart summarizing the changes of *I*_LCa_ densities at −10 mV from control, DM and DM + Ex-4 retinas. (D) Timeline of the experimental procedure and data collection. On the 17th day after STZ injection, DM rats received eye drops of Ex-4 or saline twice daily for two weeks. After 2 weeks of eye drops, all rats were sacrificed to conduct patch-clamp recording. (E) Representative *I*_LCa_ from an RGC of a DM rat treated with saline (DM + Saline) and an RGC of a DM rat treated with Ex-4 eye drops (DM + Ex-4). (F) Comparison of the I–V relationships of *I*_LCa_ obtained in RGCs from rats in DM + Saline group and DM + Ex-4 group (∗p < 0.05 by Sidak’s multiple comparisons test after two-way RM ANOVA). (G) Bar chart summarizing the changes of *I*_LCa_ densities obtained at −10 mV from DM + Saline group and DM + Ex-4 group. Data are presented as mean ± SD. See also Figure S2.

We investigated whether the *I*_LCa_ in RGCs in DM rats after 4 weeks of hyperglycemia was altered. Figure 2A shows typical *I*_LCa_ traces that were recorded in a control RGC and in a diabetic RGC before and after Ex-4 application. Plotting the I–V curves of *I*_LCa_ showed that hyperglycemia significantly and voltage-dependently enhanced *I*_LCa_ densities (p < 0.0001 vs. control, two-way RM ANOVA, Figure 2B) and that perfusion of Ex-4 significantly suppressed the *I*_LCa_ densities of diabetic RGCs (p < 0.05 vs. DM, Figure 2B). At −10 mV, *I*_LCa_ density was increased to −53.40 ± 21.96 pA/pF in DM group (p < 0.001, Figure 2C) from the control value of −36.29 ± 19.12 pA/pF. However, this increase was significantly suppressed by additional Ex-4 (39.53 ± 16.60 pA/pF, p < 0.01 vs. DM).

We further tested whether Ex-4 eye drops had an effect on diabetic RGCs by first demonstrating that Ex-4 administered via eye drops could reach the retina. We observed a significant increase in the amount of Ex-4 in the retinas 1 (0.082 ± 0.009 ng) and 2 h (0.055 ± 0.001 ng) after topical administration (Figure S2A). Ex-4 was then administered by eye drops (20 μg/kg) twice daily starting 2 weeks after the onset of DM and was prolonged for 2 more weeks (Figure 2D). Ex-4 did not reduce blood glucose levels or affect body weight in DM rats (Figures S2B and S2C), but did reduce *I*_LCa_ in diabetic RGCs (Figure 2E). The I–V curve for the DM + Ex-4 group exhibited a significant downward shift compared to the DM + Saline (vehicle) group (two-way RM ANOVA, p < 0.01, Figure 2F). The Ex-4-induced suppression of *I*_LCa_ density was observed at both −10 mV and 0 mV (p < 0.05, Figures 2F and 2G).

### Topical administration of Ex-4 promotes diabetic RGC survival by suppressing L-VGCCs

**Figure 3:**
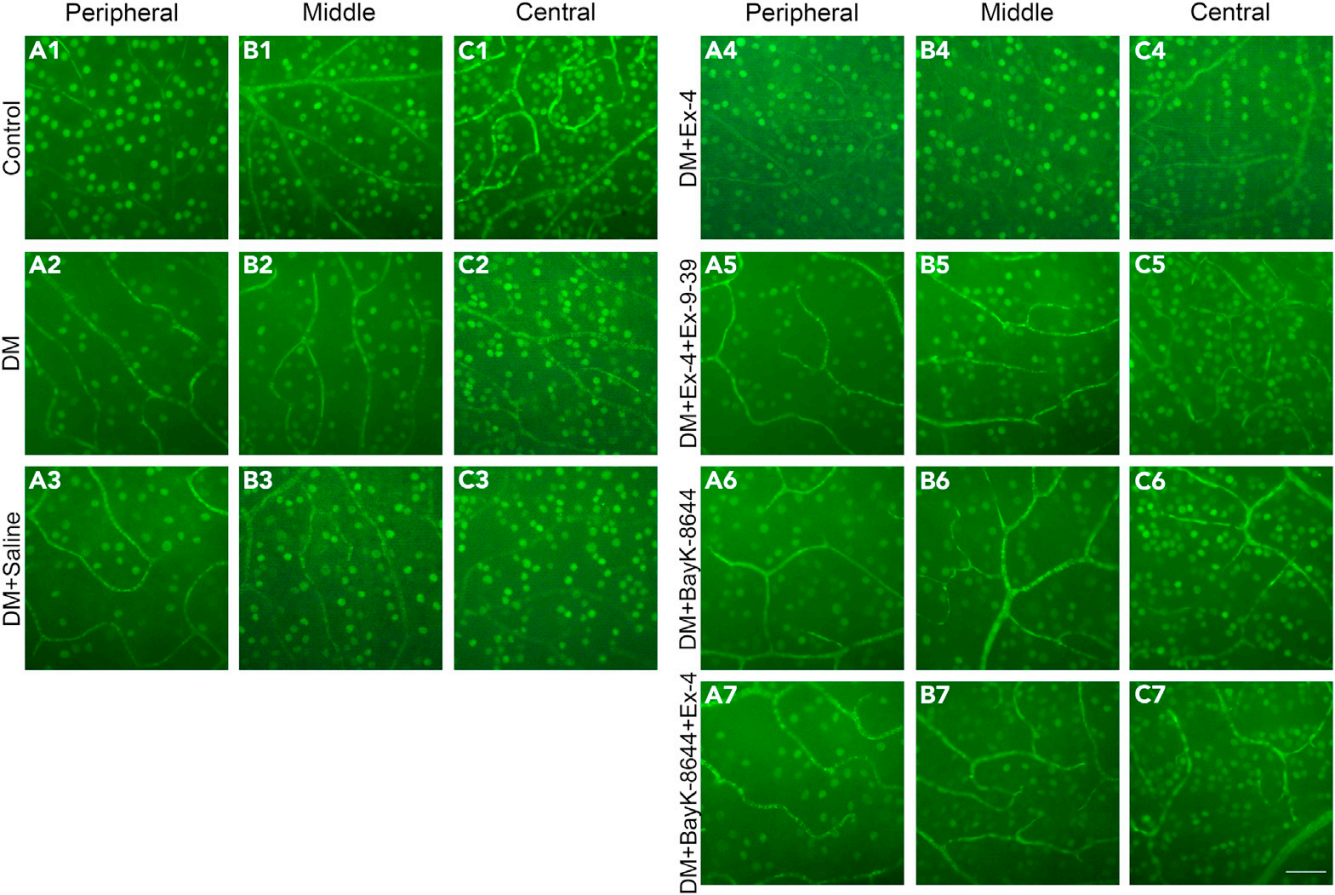
Brn3a-labeled RGCs in control, DM and different drug-treated retinas (A1–C3) Fluorescence micrographs from the peripheral, middle and central regions in flat-mount retinas showing Brn3a-labeled surviving RGCs in normal (A1–C1), DM eyes treated with (A3–C3) or without saline (A2–C2). (A4–C7) Fluorescence micrographs from the peripheral, middle and central regions in flat-mounted retinas depicting Brn3a-labeled RGCs in DM eyes treated with Ex-4 (A4–C4), Ex-9-39+Ex-4 (A5–C5), BayK-8644 (A6–C6), or BayK-8644+Ex-4 (A7–C7). Topical administration of Ex-4 increased RGC viability in the peripheral and middle regions of the retinas (A4, B4). Administration of Ex-9-39 (A5, B5) or BayK-8644 (A7, B7) blocked the protective effects of Ex-4 on RGCs. Scale bar: 50 μm.

**Figure 4:**
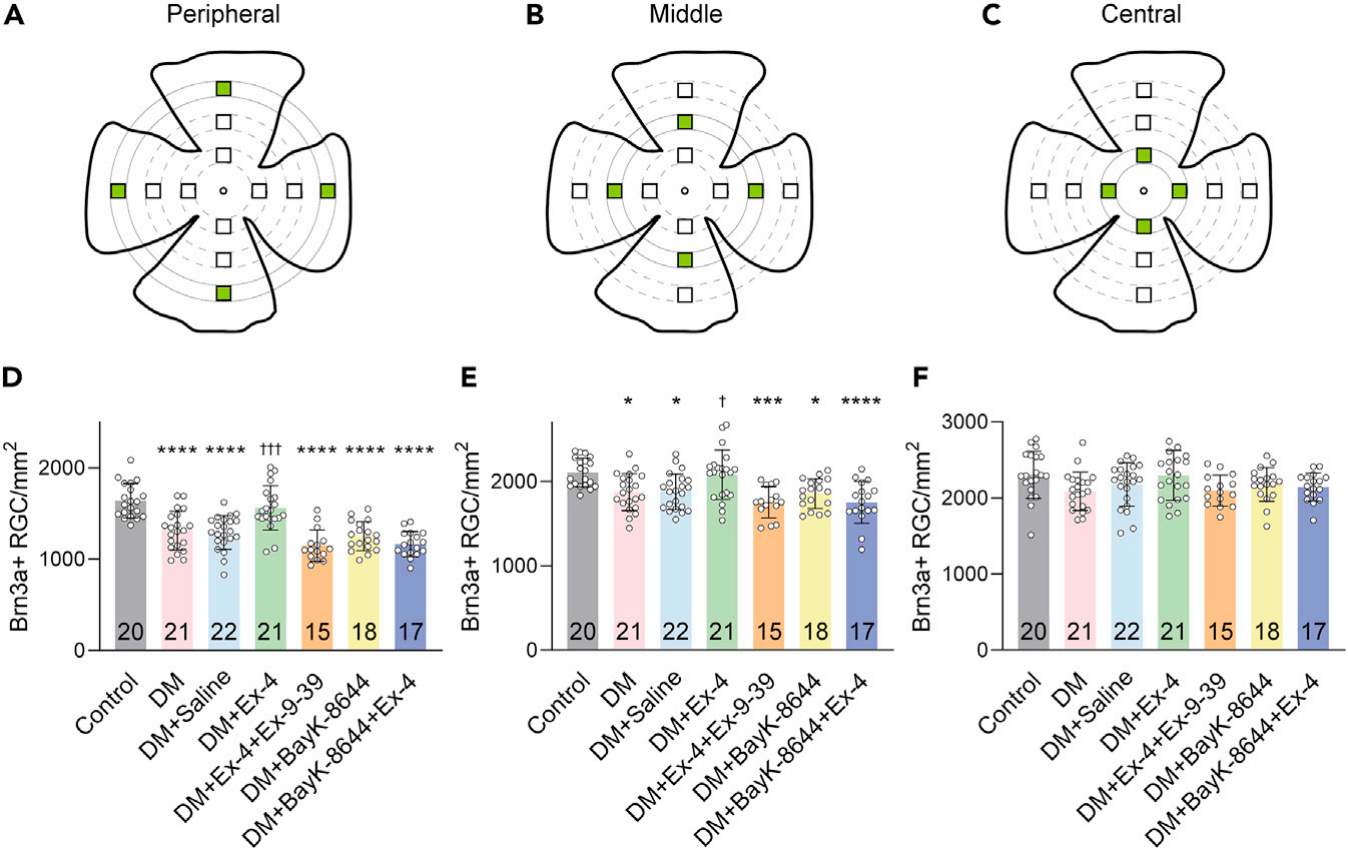
Quantitative analysis of the density of surviving RGCs (A–C) Schematic diagrams of flat-mount retinas, illustration of 12 areas of RGC quantification (4 peripheral, 4 middle and 4 central, indicated by green squares). (D–F) Quantitative analysis of the density of surviving RGCs in the peripheral (D), middle (E) and central regions (F) of retinas from control, DM eyes, saline-treated DM eyes, Ex-4-treated DM eyes, DM eyes treated with Ex-4+Ex-9-39, BayK-8644, or Ex-4+BayK-8644. Retina numbers are noted in the bars. Data are presented as mean ± SD, ∗p < 0.05, ∗∗∗p < 0.001 and ∗∗∗∗p < 0.0001 vs. control; †p < 0.05 and †††p < 0.001 vs. DM + Saline by Tukey’s multiple comparisons test after one-way ANOVA.

To determine whether the effect of Ex-4 on L-VGCCs promotes RGC survival in DM retinas, we used the antibody for Brn3a, a specific marker for RGCs,^45^ and counted the number of Brn3a-labeled (Brn3a+) RGCs in whole-mounted retinas. Saline, Ex-4, Ex-4+Ex-9-39, BayK-8644 or BayK-8644+Ex-4 was respectively administrated by eye drops twice daily for 2 weeks starting 2 weeks after the onset of DM (refer to Figure 2D); afterward, RGC survival was evaluated. Figure 3 shows the representative images of different areas of the retinas for different groups. The number of RGCs was assessed in 12 regions: 4 peripheral (Figure 4A), 4 middle (Figure 4B) and 4 central regions (Figure 4C) (for details see STAR Methods). As shown in Figures 4D and 4E, the effects of the 7 groups on the mean densities of surviving RGCs in the peripheral and middle regions were significant (one-way ANOVA, peripheral, p < 0.0001; middle, p < 0.0001). The mean densities of RGCs in the peripheral (1311 ± 212.7 cells/mm^2^) and middle regions (1873 ± 218.6 cells/mm^2^) in DM eyes were significantly lower than those in the control eyes (peripheral: 1639 ± 189.1 cells/mm^2^, p < 0.0001, Figure 4D; middle: 2107 ± 170.5 cells/mm^2^, p < 0.05, one-way ANOVA followed by Tukey’s multiple comparisons test; Figure 4E). In DM eyes, Ex-4 markedly increased RGC survival because the RGC densities in the peripheral and middle regions were 1561 ± 244.7 cells/mm^2^ and 2081 ± 295.5 cells/mm^2^ in DM + Ex-4 eyes compared with 1292 ± 184.8 cells/mm^2^ (p < 0.001, Figure 4D) and 1877 ± 212.0 cells/mm^2^ (p < 0.05, Figure 4E) in DM + Saline eyes. These data indicate that topical administration of Ex-4 significantly enhances the survival of RGCs in DM eyes.

Next, we examined whether the protective effect of Ex-4 was mediated by GLP-1R. DM rats were treated with eye drops containing Ex-4 (40 μg/kg/day) and Ex-9-39 (60 μg/kg/day). As shown in Figures 3A3, 3B3, 3A5, 3B5, 4D, and 4E, the density of RGCs in Ex-4+Ex-9-39-treated DM eyes was similar to saline-treated DM eyes in the peripheral (1148 ± 172.2 cells/mm^2^ vs. 1292 ± 184.8 cells/mm^2^, p > 0.05, Tukey’s multiple comparisons test after one-way ANOVA) and middle regions (1755 ± 185.7 cells/mm^2^ vs. 1877 ± 212.0 cells/mm^2^, p > 0.05), suggesting the involvement of GLP-1R in the Ex-4 effect. To confirm that Ex-4 exerts its effects via L-VGCCs, DM rats were treated with eye drops of Ex-4 and BayK-8644 (140 μg/kg/day), a specific L-VGCC agonist. BayK-8644 prevented the effects of Ex-4 on RGC survival, resulting in densities of 1165 ± 137.2 cells/mm^2^ in the peripheral region (p < 0.0001 vs. control, Figure 4D) and 1752 ± 249.2 cells/mm^2^ in the middle region (p < 0.0001 vs. control, Figure 4E). Ex-9-39 and BayK-8644 did not worsen the effects of DM.

Different from the peripheral and middle regions of retinas, the effects of the 7 groups on the mean densities of surviving RGCs in the central region were not significant (p > 0.05, one-way ANOVA). Although the RGC density tended to decrease due to DM, no significant difference was observed between DM (2086 ± 251.5 cells/mm^2^) and control retinas (2304 ± 309.9 cells/mm^2^, p > 0.05, Figure 4F).

In addition, we examined overall RGC density of whole retina for the significance test and found that the mean RGC density in DM retinas (1757 ± 192.9 cells/mm^2^) was significantly lower than that in control retinas (2017 ± 158.0 cells/mm^2^, p < 0.001, data not shown). Moreover, Ex-4 significantly enhances the survival of overall RGCs in DM eyes (1980 ± 257.9 cells/mm^2^ in DM + Ex-4 vs. 1782 ± 164.5 cells/mm^2^, p < 0.01, in DM + Saline).

We also measured the area of the whole retina and found no significant difference between DM (57.20 ± 3.3 mm^2^, n = 10) and control groups (56.95 ± 3.3 mm^2^, n = 9) (p > 0.05, data not shown). All these results suggest that RGC reduction mainly occurs in the peripheral and middle retinas in 4-week DM rats and that topical administration of Ex-4 has a significant protective effect on RGCs in these regions, whereas RGCs in the central region were less affected by DM.

### cAMP-PKA signaling pathway mediates Ex-4-induced suppression of *I*_LCa_

**Figure 5:**
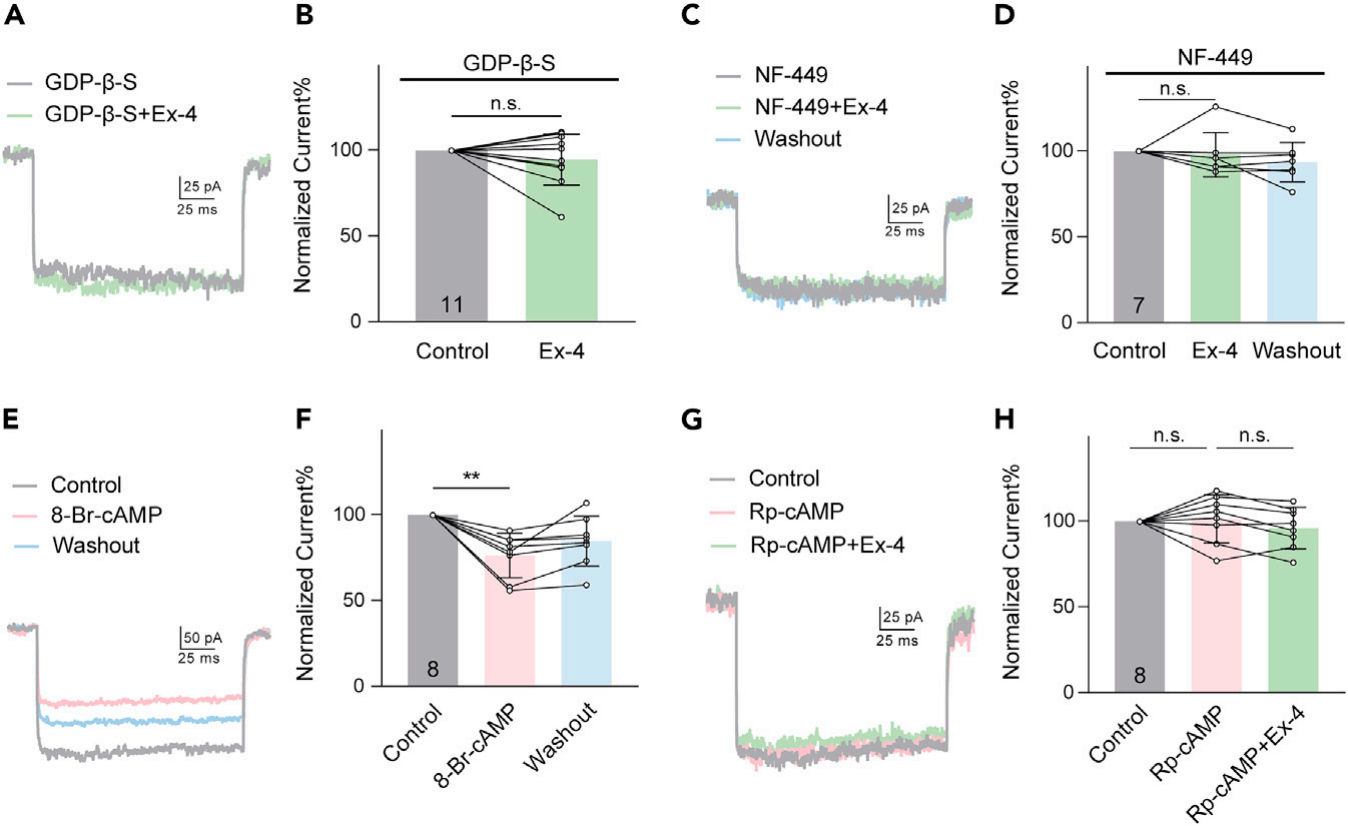
Involvement of cAMP-PKA signaling pathway in the Ex-4-induced suppression of *I*_LCa_ (A) Current traces obtained from an RGC at −10 mV showing that during internal dialysis of 3 mM GDP-β-S, addition of Ex-4 failed to suppress the *I*_LCa_. (B) Bar chart summarizing the Ex-4 effect on the amplitudes of the currents in the presence of GDP-β-S (n = 11). The data obtained for each cell were normalized to the amplitudes obtained at least 8 min after GDP-β-S infusion. (C and D) Representative recordings of an RGC showing that Ex-4 did not change the *I*_LCa_ of the RGC pre-incubated with NF-449 (10 μM), and summary data are shown in (D) (n = 7). (E and F) Current traces recorded in an RGC at −10 mV showing that perfusion of 8-Br-cAMP (500 μM) suppressed the *I*_LCa_ (E), and summary data are shown in (F) (n = 8). (G and H) Sample traces recorded from another RGC at −10 mV showing that perfusion of 50 μM Rp-cAMP blocked the Ex-4-induced reduction of the *I*_LCa_ (G), and summary data are shown in (H) (n = 8). Data are presented as mean ± SD, n.s., p > 0.05 and ∗∗p < 0.01 by paired *t* test.

Following GLP-1R activation, the main intracellular signaling pathway stimulates Gs, which in turn activates cAMP-PKA in neurons and pancreatic beta cells.^46^^,^^47^ To investigate whether this pathway is involved, we added 3 mM GDP-β-S, a nonhydrolyzable G-protein inhibitor, into patch pipettes. When the *I*_LCa_ amplitudes of RGCs reached a steady level (control), Ex-4 application did not change the *I*_LCa_ (94.73 ± 14.69% of control, p > 0.05, Figures 5A and 5B). When cell suspensions were pre-incubated with 10 μM NF-449, a Gsα protein inhibitor, for 20 min before recording, additional Ex-4 failed to change the *I*_LCa_ in RGCs (98.14 ± 12.85% of control, p > 0.05, Figures 5C and 5D). However, perfusion of 500 μM 8-Br-cAMP, a membrane-permeable cAMP analog, significantly suppressed the *I*_LCa_ to 76.53 ± 12.96% of control (p < 0.01, Figures 5E and 5F), thus mimicking the Ex-4 effect. Furthermore, applying 50 μM of RP-cAMP, a PKA inhibitor, did not change the *I*_LCa_ (101.60 ± 13.92% of control, p > 0.05, Figures 5G and 5H), and co-applying Ex-4 no longer suppressed the currents (96.22 ± 12.04% of control, p > 0.05, Figures 5G and 5H).

### Ca^2+^, calmodulin (CaM), calcineurin and protein phosphatase 1 (PP1) are involved in Ex-4-induced suppression of *I*_LCa_

**Figure 6:**
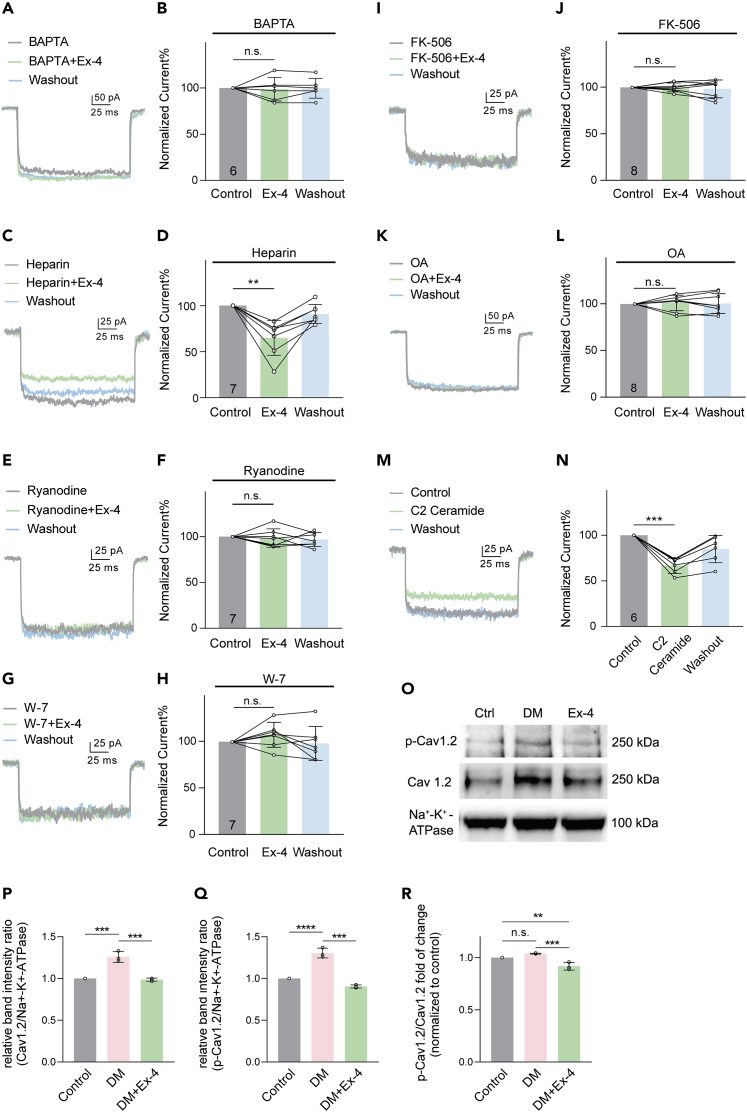
Involvement of intracellular Ca^2+^, CaM, calcineurin and PP1 in suppression of *I*_LCa_ by Ex-4 (A and B) Representative recordings from an RGC at −10 mV, showing that during internal infusion of Ca^2+^-free solution (containing 10 mM BAPTA; control), Ex-4 failed to suppress the *I*_LCa_, and summary data are shown in (B) (n = 6). (C and E) Current traces of two RGCs, showing that during internal dialysis of 5 mg/mL heparin (C), but not ryanodine (50 μM, E), the Ex-4-induced suppression of *I*_LCa_ was seen. (D and F) Bar charts summarizing the effects of Ex-4 on the *I*_LCa_ amplitudes in the presence of heparin (n = 7) (D) or ryanodine (n = 7) (F). (G, I, and K) Representative recordings obtained from three different RGCs showing that Ex-4 no longer reduced the *I*_LCa_ amplitudes during the internal infusion of 100 μM W-7 (G), 50 μM FK-506 (I) and 1 μM OA (K) to block CaM, calcineurin and PP1, respectively. (H, J, and L) Bar charts summarizing the results regarding the effects of W-7 (n = 7) (H), FK-506 (n = 8) (J) or OA (n = 8) (L). (M and N) Current traces of an RGC, showing that perfusion of C2 Ceramide (500 nM) suppressed the *I*_LCa_ (M), and summary data are shown in (N) (n = 6). Data are presented as mean ± SD, n.s., p > 0.05, ∗∗p < 0.01 and ∗∗∗p < 0.001 by paired *t* test. (O) Representative immunoblots showed changes in the expression of L-VGCC subunits Cav1.2 and *p*-Cav1.2 in membrane components in retinas from the control, DM rats treated with or without Ex-4 eye drops. Na^+^-K^+^-ATPase served as loading control. (P and Q) Densitometric analysis revealing a significant increase in Cav1.2 (P) and *p*-Cav1.2 (Q) protein levels in DM retinas. Topical administration of Ex-4 significantly decreased Cav1.2 and *p*-Cav1.2 levels in DM retinas. (R) Quantitative analysis of *p*-Cav1.2 relative to Cav1.2 total protein is shown in the bar graph. The level of *p*-Cav1.2/Cav1.2 did not change in DM retinas, but significantly decreased in the DM + Ex-4 group. n = 3 for each group. Data are presented as mean ± SD. n.s., p > 0.05, ∗∗p < 0.01, ∗∗∗p < 0.001 and ∗∗∗∗p < 0.0001 by post hoc Tukey’s multiple comparisons test after one-way ANOVA. See also Figure S3.

Since our recent work demonstrated that Ex-4 increased the intracellular Ca^2+^ concentration ([Ca^2+^]_i_) in isolated rat RGCs via PKA activation,^48^ we further investigated whether the Ex-4 effect on *I*_LCa_ in RGCs depends on [Ca^2+^]_i_. BAPTA (10 mM) was added to the recording pipette to chelate intracellular free Ca^2+^, additional Ex-4 did not suppress *I*_LCa_ in RGCs (98.67 ± 12.63% of control, p > 0.05, Figures 6A and 6B). No Ca^2+^ was present in the extracellular solution for our experiments, indicating that changes in [Ca^2+^]_i_ are potentially due to an altered Ca^2+^ release from intracellular calcium stores rather than changes in Ca^2+^ influx via Ca^2+^ channels. Inositol 1,4,5-trisphosphate (IP_3_)- and/or ryanodine-sensitive pathways mediate Ca^2+^ release from intracellular calcium stores. Ex-4 application still suppressed *I*_LCa_ in RGCs during intracellular infusion of 5 mg/mL of the IP_3_ receptor antagonist heparin (64.91 ± 19.02% of heparin alone, p < 0.01, Figures 6C and 6D). However, after internal infusion of 50 μM ryanodine, which depletes ryanodine-sensitive Ca^2+^ sites, Ex-4 no longer suppressed the *I*_LCa_ (98.57 ± 10.05% of ryanodine alone (p > 0.05, Figures 6E and 6F).

Previous studies showed that activating cAMP and/or PKA suppressed *I*_LCa_ in neurons.^49^^,^^50^ However, these suppressing effects may be indirect because Ca^2+^ has been shown to activate calcineurin by binding CaM, which relieves PP1 inhibition, and then PP1 inactivates L-VGCCs by dephosphorylating them.^51^ We tried to test whether this signaling pathway was also involved in the Ex-4 effect on *I*_LCa_. As shown in Figures 6G and 6H, after intracellular application of the CaM inhibitor W-7 (100 μM) for about 8 min, *I*_LCa_ reached a steady level (control), and extracellular perfusion of Ex-4 failed to suppress the *I*_LCa_ of RGCs (107.30 ± 13.40% of control). Similarly, during intracellular application of the calcineurin inhibitor FK-506 (50 μM) or the PP1 inhibitor okadaic acid (OA, 1 μM), adding Ex-4 no longer reduced the *I*_LCa_ in RGCs (99.99 ± 4.76% of control for FK-506, 101.30 ± 8.31% of control for OA, p > 0.05 for both, Figures 6I–6L). Moreover, applying the PP1 activator C2 Ceramide (500 nM) suppressed *I*_LCa_ to 66.5 ± 8.41% of control (p < 0.001, Figures 6M and 6N). These results suggest the involvement of the CaM/calcineurin/PP1 signaling pathway in the Ex-4 effect.

We further investigated whether Ex-4 could alter L-VGCC phosphorylation in DM retinas. We examined changes in expression levels for Cav1.2, a main subunit of L-VGCCs expressed in rat RGCs. Western blotting showed that the levels of Cav1.2 total protein and *p*-Cav1.2 protein in DM retinas increased considerably to 125.90 ± 6.45% (one-way ANOVA, p < 0.001, Figures 6O and 6P) and 130.50 ± 5.89% of control (p < 0.0001, Figures 6O and 6Q), respectively. Applying Ex-4 eye drops for 2 weeks abolished the DM-induced upregulation of Cav1.2 and *p*-Cav1.2 (p < 0.001 vs. DM, Figures 6P and 6Q). Although the *p*-Cav1.2/Cav1.2 levels did not change in DM retinas (103.90 ± 0.39% of control, p > 0.05, one-way ANOVA, Figures 6O and 6R), Ex-4 eye drops significantly decreased the phosphorylation of Cav1.2 in the DM retinas (88.56 ± 3.73% of DM, p < 0.001, Figure 6R), suggesting that Ex-4 inactivates L-VGCCs by dephosphorylating Cav1.2.

Although GLP-1R has also been shown to activate the downstream phospholipase C (PLC)/PKC signaling pathway,^52^ our results did not support the involvement of this pathway in the Ex-4 effect on *I*_LCa_ in RGCs. As shown in Figures S3A and S3B, during internal infusion of 10 μM U-73122, an inhibitor of PLC, perfusion of Ex-4 still suppressed the *I*_LCa_ to 71.44 ± 6.11% of control (n = 6, p < 0.0001). Moreover, in the presence of 10 μM Bis-Ⅳ, a PKC inhibitor, Ex-4 also significantly suppressed the currents (58.19 ± 9.98% of control, n = 6, p < 0.001; Figures S3C and S3D).

### Ex-4 ameliorates the functional changes in RGCs induced by DM

**Figure 7:**
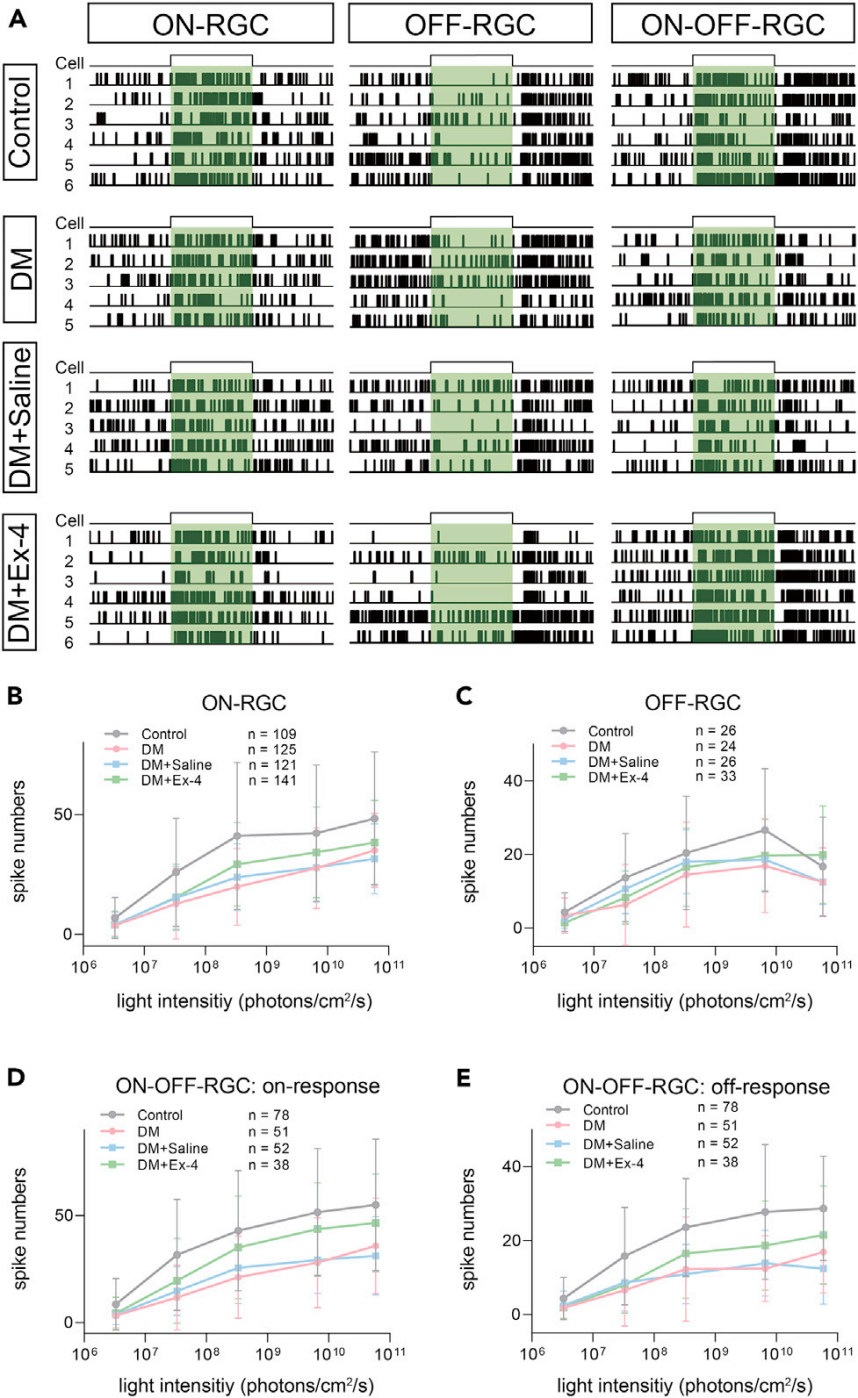
Quantitative analysis of light responses of RGCs in different experimental groups (A) Representative raster plots for light-evoked spiking activity of RGCs obtained via MEA recording on whole-mount retinas in different conditions. The spiking activities of ON-, OFF-, and ON-OFF-RGCs in response to full-field, 500 nm (1-s) light pulses with the intensity of 5.86×10^10^ photons/cm^2^/s (green bars) were obtained from three control, three DM, three DM retinas treated with eye drops of saline (DM + Saline) and three DM retinas treated with eye drops of Ex-4 (DM + Ex-4). Each trace represents the spike train from a clearly distinguishable single unit identified by offline spike sorting. (B) Group data comparing irradiance–response (I–R) functions of total spike number of light response of ON-RGCs in control, DM, DM + Saline and DM + Ex-4 retinas. Note that DM rats (n = 125 cells) exhibited a significantly decreased total spike number as compared to those in control eyes (n = 109, p < 0.0001 by two-way RM ANOVA). Topical administration of Ex-4 (n = 141) significantly increased the total spike number in DM retinas (p < 0.001 vs. DM). (C) Group data comparing I–R curve of total spike number of OFF-RGCs in four groups. Note that the I–R curve in the DM group (n = 24) shifted downward significantly compared to that in control (n = 26, p < 0.05 by two-way RM ANOVA). No significant changes were detected in the DM rats treated with Ex-4 (n = 33, p > 0.05 vs. DM). (D) Group data comparing I–R curve of total spike number of ON-OFF-RGCs during light stimulation (on-response) in control, DM, DM + Saline and DM + Ex-4 retinas. Note that the I–R curve in DM eyes (n = 51) shifted downward significantly compared to that in control eyes (n = 78, p < 0.0001 by two-way RM ANOVA). Topical administration of Ex-4 significantly increased the total spike number in DM eyes (n = 38, p < 0.01 vs. DM). (E) Group data comparing I–R curve of total spike number of ON-OFF-RGCs after light stimulation (off-response) in four groups. Note that the I–R curve in DM eyes shifted downward significantly compared to that in control eyes (p < 0.0001 by two-way RM ANOVA). Topical administration of Ex-4 significantly increased the total spike number in DM eyes (p < 0.01 vs. DM). Data are presented as mean ± SD.

To examine whether DM causes functional abnormalities in RGCs, we performed high-throughput evaluations of light responses of RGCs using the multielectrode array (MEA) technique. Figure 7A shows the typical raster graphics of light-evoked spikes from the ON-, OFF- and ON-OFF-RGCs at a light intensity of 5.86×10^10^ photons/cm^2^/s recorded by MEA in different groups. Light-evoked spike numbers increased as a function of light intensity in ON-RGCs in the control, DM, DM + Saline and DM + Ex-4 groups. In the DM groups, the total spike numbers of ON-, OFF- and ON-OFF-RGCs significantly decreased compared to those in corresponding control groups [two-way RM ANOVA, p < 0.0001 for ON- and ON-OFF-RGCs (for both on- and off-responses); p < 0.05 for OFF-RGCs, Figures 7B–7E], as evidenced by the significant downward scaling of the irradiance–response (I–R) curves, suggesting a decrease in photoresponse. However, the I–R curves of ON- and ON-OFF-RGCs in the DM + Ex-4 group significantly shifted upward compared to the DM group [p < 0.001 for ON-RGCs; p < 0.01 for ON-OFF-RGCs (for both on- and off-responses), Figures 7B, 7D, and 7E], suggesting an increased response gain due to Ex-4 administered by eye drops. In contrast, the I–R curve of OFF-RGCs in the DM + Ex-4 group was not significantly different from the DM group (p > 0.05, Figure 7C), indicating that Ex-4 does not work. No significant differences in total spike number were detected between the DM group and the DM + Saline group for the three types of RGCs (p > 0.05).

## Discussion

In this work, we first show that 4 weeks of hyperglycemia resulted in downregulation of rat retinal GLP-1 mRNA levels and upregulation of *I*_LCa_ density in RGCs. Second, Ex-4 eye drops prevented upregulations of *I*_LCa_ densities and retinal L-VGCC protein expression induced by hyperglycemia. Third, Ex-4 eye drops promoted RGC survival and its protective effects could be blocked by the competitive GLP-1R antagonist Ex-9-39 or the selective L-VGCC agonist BayK-8644. Fourth, Ex-4 significantly reduced *I*_LCa_ in RGCs and the inhibitory role could be mediated by exciting the GLP-1R/Gs/cAMP-PKA/ryanodine/Ca^2+^/CaM/calcineurin/PP1 signaling pathway. Fifth, Ex-4 decreased phosphorylation of Cav1.2 in DM retinas. Sixth, Ex-4 improved the light-evoked spiking ability of ON- and ON-OFF-RGCs in DM rats. Our results established a mechanism by which activation of GLP-1R protects damaged RGCs via modulating L-VGCCs. A schematic diagram of possible mechanisms is shown in (Figure S4). These results suggest that L-VGCCs may be a therapeutic target for inhibiting early DR and topical administration of GLP-1R agonists could provide a noninvasive and effective approach in the treatment of the retinopathy independently of their hypoglycemic action.

Intracellular Ca^2+^ overload mediated through VGCCs has been associated with neuronal degeneration in glaucoma patients and animal models, and in rat retinal ischemic models.^20^^,^^21^^,^^22^^,^^23^ This is the first work to show an upregulation of *I*_LCa_ densities in diabetic RGCs, suggesting an increased Ca^2+^ influx in RGCs at the early stage of DM. This is consistent with a previous study on dorsal horn neurons that showed an increase in activity for L-VGCCs in DM rats.^53^ Moreover, the expression levels of the Cav1.2 subunit in the DM retinas were significantly increased, which may be one of the reasons for the increase of *I*_LCa_ in RGCs. GLP-1 and GLP-1R were expressed in the vertebrate retinas,^29^^,^^30^^,^^31^^,^^32^^,^^33^^,^^34^^,^^35^^,^^36^ suggesting that the GLP-1 system plays an important role in the regulation of visual function. Actually, our recent study showed that GLP-1/Ex-4 significantly suppresses GABA receptor-mediated currents in rat RGCs through GLP-1R activation.^48^ Here we further found that perfusion of Ex-4 significantly suppressed *I*_LCa_ in normal and diabetic RGCs and Ex-4 eye drops also reversed the upregulation of *I*_LCa_ densities and expression levels of Cav1.2 induced by hyperglycemia. GLP-1/Ex-4 was consistently shown to suppress Ca^2+^ currents in cultured hippocampal neurons and hypothalamic neurons.^37^^,^^54^ In contrast to these results, GLP-1 enhanced *I*_LCa_ in canine cardiomyocytes.^55^ These data suggest that the GLP-1 effect on the Ca^2+^ currents is cell-type-dependent.

We demonstrated that RGC density decreased significantly in the peripheral and middle retinal regions in 4-week DM rats, but decreased more in the peripheral region. These results were consistent with previous studies showing RGC loss in STZ-induced 4-week diabetic rats.^5^^,^^56^ RGC density in the central retinal region of DM rat also showed a decreasing trend. These results suggest that RGC death caused by hyperglycemia starts in the peripheral retina and gradually develops to the central retina. Similar damage also occurred in Ins2^Akita^ DM mice, with RGC loss being detected in the peripheral retina after 3 months of hyperglycemia but not in the central retina.^4^ Previous study showed that GLP-1 mRNA and protein were downregulated in retinas from donors with DM.^29^ GLP-1 has been shown to cross the blood-brain barrier to exert effective effects in the central nervous system.^38^^,^^41^ However, whether GLP-1 can cross the blood-retinal barrier remains unknown. But since the endogenous GLP-1 has a very short half-life in plasma (only about 1–2 min),^26^^,^^57^ due to degradation by the enzyme dipeptidyl peptidase-4,^26^^,^^44^^,^^58^^,^^59^^,^^60^^,^^61^ the amount of GLP-1 that can reach the retina through blood circulation may be limited. If GLP-1 does not cross the blood-retinal barrier, systemic GLP-1 could not activate its receptors within the retina. In addition, since GLP-1 is expressed by retinal neurons, it is reasonable to assume that GLP-1R in the retina respond to local release of GLP-1. We also identified a lower level of GLP-1 mRNA in DM rat retinas and Ex-4 eye drops reversed the RGC loss in DM rats without any effect on blood glucose levels. These results strongly suggest that Ex-4 has a direct neuroprotective effect that is independent of its ability to increase insulin secretion or lower blood glucose levels. Previous studies have also shown that systemic or local administration of GLP-1 or GLP-1R agonists reduces the loss of retinal neurons in diabetic animals.^12^^,^^29^^,^^33^ However, the mechanisms underlying the neuroprotection induced by GLP-1R agonists are not fully understood. We found that the protective effect of Ex-4 on diabetic RGCs was blocked by either the GLP-1R antagonist Ex-9-39 or the L-VGCC agonist BayK-8644, suggesting that the Ex-4 effect on RGCs is mediated by GLP-1R and L-VGCCs. In this regard, it has been shown that administering calcium channel blockers promotes RGC survival in patients and animal models with glaucoma^20^ and in a retinal ischemic rat model.^23^ All of these data suggest that Ex-4 promotes RGC survival by reducing intracellular Ca^2+^ overload by inhibiting L-VGCCs of RGCs in DM retinas. In addition, previous studies showed that the protective mechanisms of GLP-1R activation on DM retinal neurons included increased prosurvival signals and decreased proapoptotic signals, proinflammatory cytokines and extracellular glutamate, as well as antioxidant effects.^12^^,^^29^^,^^33^^,^^62^

Through pharmacological dissections, we demonstrated that a distinct cAMP-PKA signaling pathway following GLP-1R activation may be responsible for the Ex-4 effect on *I*_LCa_ in RGCs. This signaling pathway in GLP-1R-mediated effects has been demonstrated in both hippocampal neurons and pancreatic beta cells.^46^^,^^47^ However, the pathway by which PKA activation inhibited *I*_LCa_ remains unknown. Our recent study showed that Ex-4-induced PKA activation causes an increase in [Ca^2+^]_i_ in rat RGCs.^48^ The elevation of [Ca^2+^]_i_ induced by Ex-4 was potentially the result of increased Ca^2+^ release from ryanodine receptor-mediated intracellular stores. Activating cAMP and/or PKA has been shown to inhibit *I*_LCa_ in tiger salamander rod and rat pinealocytes.^49^^,^^50^ PKA activation may indirectly suppress *I*_LCa_ through the Ca^2+^/CaM/calcineurin/PP1 signaling pathway, in which PP1 catalyzes the dephosphorylation of L-VGCCs, curtailing Ca^2+^ entry.^51^ This pathway may also underlie the Ex-4 effect on *I*_LCa_ because the Ex-4-induced inhibition of *I*_LCa_ no longer occurred when CaM, calcineurin and PP1 were respectively blocked.

Furthermore, we found that Ex-4 eye drops significantly decreased *p*-Cav1.2/Cav1.2 levels in the DM retinas, suggesting Ex-4 inactivates L-VGCCs by dephosphorylating Cav1.2, thereby reducing Ca^2+^ influx.

MEA recordings showed that the light-induced total spike number of ON-, OFF- and ON-OFF-RGCs were significantly decreased in 4-week DM rats, indicating that DM has a significant impact on overall RGC-mediated retinal outputs to higher centers. The ON and OFF signaling pathways and their interactions in the visual system were reported to be closely related to contrast detection^63^; therefore, it is reasonable to speculate that declined visual contrast sensitivity, which has been considered an early sign of neural retinal dysfunction in diabetic patients and animals,^64^^,^^65^^,^^66^ may be related to the diminished light responses of RGCs that we observed. Notably, Ex-4 eye drops significantly increased the light responses of ON- and ON-OFF-RGCs, but not OFF-RGCs.

It should be noted that some clinical trials demonstrated beneficial effects of GLP-1R agonists by oral or intravenous injection in cardiovascular outcomes but found that they significantly increased the rate of severe DR complications.^34^^,^^67^^,^^68^^,^^69^ Moreover, initial worsening of DR has been reported to be likely the consequence of rapid improvement of hyperglycemia (large reduction of HbA1c/rapid improvement of blood glucose levels).^34^^,^^70^^,^^71^ However, a recent paper shows that the early worsening of DR by rapid optimization of blood glucose levels is not a clinical problem in patient with mild or moderate DR.^72^ The development of new techniques for retinal imaging and functional assessment will help us to detect early changes and further to clarify whether therapies based on GLP-1R activation are good or bad for the human diabetic retina.^73^

### Limitations of the study

We did not further investigate the mechanism by which Ex-4 improved the light-evoked spiking ability of RGCs in DM rats. Previous studies demonstrated that GLP-1R immunostaining exists in the GCL and that there are weak positive signals in the inner nuclear layer and inner plexiform layer of retina,^29^^,^^30^^,^^32^^,^^33^^,^^34^^,^^35^^,^^36^ suggesting that Ex-4 may also play a neuroprotective role for the presynaptic cells of RGCs, such as amacrine cells and/or bipolar cells, whose somata are located in the inner nuclear layer and processes in the inner plexiform layer. However, the exact sites and mechanisms of these protective effects need to be explored further.

## STAR★Methods

### Key resources table

**Table:** 

REAGENT or RESOURCE	SOURCE	IDENTIFIER
**Antibodies**		

Rabbit polyclonal anti-Cav1.2 (CACNA1C)	Alomone	Cat#ACC-003; RRID: AB_2039771
Rabbit polyclonal anti-CACNA1C (Phospho-Ser1981)	Signalway Antibody	Cat#12674
Peroxidase AffiniPure Donkey Anti-Rabbit IgG (H+L)	Jackson Labs	Cat#711-035-152; RRID: AB_10015282
Donkey polyclonal anti-Mouse IgG (H+L) Highly Cross-Adsorbed Secondary Antibody, Alexa Fluor™ Plus 488	Invitrogen	Cat#A32766; RRID: AB_2762823
Mouse polyclonal anti-Brn3a	Millipore	Cat#MAB1585; RRID: AB_94166
Sodium Potassium ATPase antibody	Abcam	Cat#ab76020; RRID: AB_1310695

**Chemicals, peptides, and recombinant proteins**		

NF-449	Cayman Chemical	Cat#13324
Exendin-4	MedChemExpress	Cat#HY-13443
Exendin-9-39	MedChemExpress	Cat#HY-P0264
(S)-(-)-Bay-K-8644	MedChemExpress	Cat#HY-15124
Nimodipine	MedChemExpress	Cat#HY-B0265
PEG-400	MedChemExpress	Cat#HY-Y0873A
C2 Ceramide	Santa Cruz	Cat#3102-57-6
Streptozocin	Sigma-Aldrich	Cat#S0130
Protease inhibitor cocktail	Sigma-Aldrich	Cat#P8340
Papain	Sigma-Aldrich	Cat#P3375
L-Cysteine	Sigma-Aldrich	Cat#168149
Ames’ medium	Sigma-Aldrich	Cat#A1420
Bis-IV	Sigma-Aldrich	Cat#B3306
FK-506	Sigma-Aldrich	Cat#F4679
OA	Sigma-Aldrich	Cat#09381
DMSO	Sigma-Aldrich	Cat#D5879
8-Br-cAMP	Sigma-Aldrich	Cat#B5386
GDP-β-S	Sigma-Aldrich	Cat#G7637
BAPTA	Sigma-Aldrich	Cat#A4926
Heparin	Sigma-Aldrich	Cat#H4784
Rp-cAMP	Sigma-Aldrich	Cat#A165
HEPES	Sigma-Aldrich	Cat#H3375
EGTA	Sigma-Aldrich	Cat#E4378
Phosphocreatine	Sigma-Aldrich	Cat#P1937
Adenosine 5′-triphosphate magnesium salt	Sigma-Aldrich	Cat#A9187
Guanosine 5′-triphosphate sodium salt hydrate	Sigma-Aldrich	Cat#G8877
D-(+)-Glucose	Sigma-Aldrich	Cat#G8270
Tetraethylammonium chloride	Sigma-Aldrich	Cat#T2265
TTX	Tocris Bioscience	Cat#1078
W-7	Tocris Bioscience	Cat#0369
U-73122	Tocris Bioscience	Cat#1268
Ryanodine	Tocris Bioscience	Cat#1329

**Experimental models: Organisms/strains**		

Sprague Dawley Rat	Shanghai Slac Laboratory Animal Co., Ltd	N/A

**Oligonucleotides**		

Primer for GLP-1: Forward: 5′-GGAGGGCCAGGCAGCAAAGG-3′	Invitrogen	N/A
Primer for GLP-1: Reverse: 5′-TCTGCGCCCAAGTTCCTCAGC-3′	Invitrogen	N/A
Primer for B2M: Forward: 5′-ATCTTTCTGGTGCTTGTCTCT-3′	Invitrogen	N/A
Primer for B2M: Reverse: 5′-TGAGGTGGGTGGAACTGAGA-3′	Invitrogen	N/A

**Software and algorithms**		

Prism 8	GraphPad Software	https://www.graphpad.com/scientificsoftware/prism/
ImageJ	NIH	https://imagej.nih.gov/ij/RRID:SCR_003070
Adobe Photoshop CS5	Adobe	https://www.adobe.com/products/photoshop.html
Pulse 8.65	HEKA Elektronik	http://www.heka.com
Pulsefit 8.62	HEKA Elektronik	http://www.heka.com
MC_Rack	Multi Channel System	https://www.multichannelsystems.com/software/mc-rack
Offline Sorter V3	Plexon	https://plexon.com/products/offline-sorter/
Image Lab	BioRad	https://www.imagelab.co/
Excel 2016	Microsoft	https://www.microsoft.com/en-us/microsoft-365/excel

**Other**		

EPC 9/2	HEKA Elektronik	N/A
USB-MEA60-Inv-BC-System	Multi channel Systems	N/A

### Resource availability

#### Lead contact

Further information and requests for resources and reagents should be directed to and will be fulfilled by the lead contact, Dr. Yong-Mei Zhong (ymzhong@fudan.edu.cn).

#### Materials availability

This study did not generate new unique reagents.

### Experimental model and study participant details

#### Animals

Animal protocols were consistent with the National Institute of Health Guide for the Care and Use of Laboratory Animals (NIH Publications No. 80-23) revised 1996, and were approved by the Animal Care and Use Committees of Fudan University. Male Sprague Dawley rats aged 4–5 weeks were purchased from SLAC Laboratory Animal Company (Shanghai, China). Since males are more susceptible to STZ than females, only male rats (which have a higher percentage of diabetes) were used to minimize the number of animals used. Rats were housed in a temperature-controlled room (25 ± 1°C) under a 12:12-hour light/dark cycle, with food and water supplied *ad libitum*. All efforts were made to minimize animal suffering and to reduce the number of animals used.

### Method details

#### Induction of diabetic rat model

Diabetes was induced in rats aged 4–5 weeks by a single intraperitoneal injection of STZ (70 mg/kg body weight, Sigma-Aldrich Corp., St. Louis, MO, USA) freshly dissolved in a sodium citrate buffer (0.1 M, pH 4.2) after fasting for 12 hours. Age-matched control rats received an equal volume of sodium citrate buffer. The blood glucose levels and body weight were measured before injection, 3 days after the STZ injection and once a week thereafter for 4 weeks (Figure S1). Rats with blood glucose levels exceeding 16.7 mM were considered to be diabetic.^74^^,^^75^

#### Real-time quantitative reverse transcription PCR

Rats were deeply anesthetized with an intraperitoneal injection of 25% urethane (10 mL/kg). Retinas were harvested, and total RNA (200 ng) was extracted and reverse-transcribed using commercially available kits (Takara Bio Inc., Shiga, Japan) according to the manufacturer’s instructions. Quantitative RT-PCR was performed using TB Green Premix Ex Taq II (Takara Bio Inc.) on the Applied Biosystems QuantStudio 3 96-Well 0.2-mL Block. The primer sequences of GLP-1 and beta-2-microglobulin (B2M) were as follows: 5′-GGAGGGCCAGGCAGCAAAGG-3′(forward) and 5′-TCTGCGCCCAAGTTCCTCAGC-3′(reverse),^76^ 5′- ATCTTTCTGGTGCTTGTCTCT-3′(forward) and 5′- TGAGGTGGGTGGAACTGAGA-3′(reverse),^77^^,^^78^ respectively. Quantitative PCR results were analyzed using the 2^–ΔΔCt^ method^79^^,^^80^: Fold change = 2^–ΔΔCt^ and ΔΔCt = (Ct_1_ – Ct_2_) – (Ct_3_ – Ct_4_). Ct_1_ and Ct_2_ denoted the critical number of cycles for the target gene (GLP-1) and the housekeeping gene (B2M) respectively in the sample of a diabetic rat. Ct_3_ and Ct_4_ denoted the critical number of cycles for the target gene (GLP-1) and the housekeeping gene (B2M) in the sample of a control rat.

#### Topical ocular treatment

Referring to the previous study,^29^ eye drops with drugs or vehicle (0.9% sodium chloride) were administered directly onto the superior corneal surface of both eyes using a micropipette after 2 weeks of hyperglycemia (Figure 2D). Some rats received eye drops (4–6 μL) of Ex-4 (40 μg/kg/day), Ex-9-39 (60 μg/kg/day)+Ex-4, BayK-8644 (140 μg/kg/day) or BayK-8644+Ex-4. The treatment was repeated twice daily for 14 days. The age-matched control rats received eye drops of saline. At day 31 post-STZ, the rats were killed by intraperitoneal injection of 25% urethane (10 mL/kg) and the eyes were enucleated. The separated retinas were used for patch-clamp recording, western blotting and immunofluorescent labeling.

#### Enzyme linked immunosorbent assay (ELISA)

To estimate whether the topical administration of Ex-4 reaches the retina, Ex-4 concentration in the retinas of normal rat eyes 1 or 2 hours after administration of Ex-4 with eye drops (20 μg/kg) was evaluated using ELISA. The separated retinas were freshly dissected and sonicated in a lysis buffer containing 20 mM Tris (pH7.5), 150 mM NaCl, 1% Triton X-100, and a protease inhibitor cocktail (P8340, Sigma-Aldrich). The level of Ex-4 was determined by Exendin-4 (Heloderma suspectum) Enzyme Immunoassay Kit (Phoenix Pharmaceuticals, Inc., Burlingame, CA, USA) following the manufacturer’s instructions.

#### Membrane protein extraction and western blot analysis

Western blot analysis was performed as described previously with minor modification.^75^ In brief, membrane protein was extracted from four retinas (as one sample) using a membrane protein extraction kit (K268, Biovision Inc., Milpitas, CA, USA). Membrane protein concentrations were determined using a standard bicinchoninic acid (BCA) assay kit (23235, Thermo Fisher Scientific, Waltham, MA, USA). Equivalent amounts of freshly extracted samples were loaded (for the detection of p-Cav1.2, the loading sample volume was 3 times that of the detection of Cav1.2, due to the low content of p-Cav1.2), subjected to 8% SDS-PAGE, and then transferred onto PVDF membranes (Immobilon-P, Millipore Co. Bedford, MA, USA). The blots were blocked for 2 hours at room temperature in a blocking solution consisting of 20 mM Tris-HCl (pH 7.4), 137 mM NaCl, 0.1% Tween-20 and 3% bovine serum albumin, and then in the same solution containing the antibody against Cav1.2 (1:500 dilution, ACC-003, Alomone, Jerusalem, Israel) or p-Cav1.2 (1:200 dilution, 12674, Signalway Antibody, Greenbelt, MD, USA) overnight at 4°C. Na^+^-K^+^-ATPase (1:20000 dilution, ab76020, Abcam, Cambridge, UK) was used as a loading control. After that, the blots were incubated in horseradish peroxidase-conjugated donkey anti-rabbit IgG secondary antibody (1:2000 dilution, Jackson Labs, West Grove, PA, USA) for 2 hours at room temperature. The immunosignals were visualized with enhanced chemiluminescence (Thermo Fisher Scientific) and analyzed on ChemiDoc XRS System with Image Lab software (Bio-Rad, Hercules, CA, USA).

#### Labeling and counting of RGCs

The procedures of immunocytochemistry refer to our previous work,^81^ with minor modifications. In brief, isolated retinas from both eyes were immediately fixed in fresh 4% paraformaldehyde in 0.1 M phosphate buffer (pH 7.4) for 2 hours at room temperature. Then the whole-mount retinas were blocked in 0.1 M phosphate buffered-saline (PBS, pH 7.4) containing 6% donkey serum and 1% Triton X-100 for 2 hours at room temperature. Mouse anti-Brn3a antibody (1:50 dilution, 3 days at 4°C, Millipore) was used to label RGCs.^45^ Immunoreactivity was detected with donkey anti-mouse IgG tagged with Alexa Fluor 488 (1:200 dilution, 2 hours at room temperature, Invitrogen, Carlsbad, CA, USA).

The number of Brn3a-positive (Brn3a+) nuclei was counted by an investigator who was blinded to the study treatments using a fluorescence microscope (Axioskop 40, Carl Zeiss Inc., Oberkochen, Germany) under a 40× objective lens. Each retina was equally divided into 4 quadrants including nasal (N), dorsal (D), temporal (T) and ventral (V) under a dissection microscope. As shown in Figures 4A–4C, twelve 256 × 256 μm areas (centered at 1, 2 and 3 mm from the optic nerve head in four quadrants) per whole mount were imaged and cell counting was conducted with ImageJ software. RGC densities (cells/mm^2^) were grouped by retinal eccentricity (central, middle and peripheral) and expressed as the mean ± SD. For the measurement of the retinal area, a series of photomicrographs for each retina was captured automatically by a fluorescence microscope (Eclipse Ni-U, Nikon, Tokyo, Japan) under a 20× objective lens, and the whole retina image was digitally reconstructed by NIS-Elements imaging software (Nikon). The whole-retina area was delineated using the Polygon tool in ImageJ software.

#### Retrograde labeling of RGCs

The detailed procedure refers to our previous work.^48^^,^^82^ Briefly, after the rats were deeply anaesthetized, 20% rhodamine-labelled fluorescent latex microsphere (LumaFluor, Durham, NC, USA) was injected into the superior colliculus bilaterally. Following a survival time of 2–3 days, RGCs were labelled clearly for electrophysiological recording.

#### Whole-cell patch-clamp recording

RGCs were acutely dissociated from retinas by enzymatic and mechanical treatments as described previously with minor modifications.^48^ In brief, isolated retinas were incubated in oxygenated Hank’s solution containing (in mM) NaCl 137, NaH_2_PO_4_ 1, NaHCO_3_ 0.5, KCl 3, CaCl_2_ 2, MgSO_4_ 1, HEPES 20, sodium pyruvate 1 and glucose 16, adjusted to pH 7.4 with NaOH and osmolality to 310–320 mOsm/L with sucrose. The retinas were then digested in 20 mL of Hank’s solution containing 40 mg papain (P3375, Sigma-Aldrich) and 4 mg L-Cysteine (168149, Sigma-Aldrich) for 28 minutes at 34°C and were mechanically dissociated with fire-polished Pasteur pipettes in Ringer’s solution containing (in mM) NaCl 135, KCl 3, CaCl_2_ 2, MgCl_2_ 1, HEPES 10, glucose 11 and sucrose 10; pH adjusted to 7.4 with NaOH and osmolality to 310–320 mOsm/L with sucrose. The cell suspension was plated onto a culture dish and observed with an inverted microscope (IX 70; Olympus Optical, Tokyo, Japan). Rhodamine-labelled RGCs, showing red fluorescence with relatively large somata (15–25 μm in diameter) with a fractured axon, were targeted for whole-cell patch-clamp recording within 2–3 hours after dissociation.

For recording Ca^2+^ currents, isolated cells were perfused with Ba^2+^ Ringer’s solution, in which 5 mM Ba^2+^ was used to substitute for Ca^2+^, containing (in mM) NaCl 120, KCl 2.5, BaCl_2_ 5, CsCl 5, HEPES 15, TEA-Cl 15, glucose 10 and tetrodotoxin (TTX) 0.4 mM, adjusted to pH 7.4 with NaOH.^83^ Ba^2+^ can go through Ca^2+^ channels as a current carrier, thus making the currents larger in size without modifying the intracellular Ca^2+^-dependent processes.^84^ Patch pipettes were made by pulling borosilicate glass capillaries (BF150-86-10, Sutter Instrument Co., Novato, CA, USA) on a micropipette puller (P-97, Sutter Instrument Co.). The pipettes (4–6 MΩ) were filled with internal solution containing (in mM): CsCl 128, CaCl_2_ 1, MgCl_2_ 2, EGTA 10, HEPES 10, ATP-Mg 2, GTP-Na 0.4, phosphocreatine 10, adjusted to pH 7.2 with CsOH and osmolality to 290–300 mOsm/L with sucrose. Whole-cell membrane currents of RGCs were recorded using a patch amplifier (EPC 9/2; HEKA Elektronik, Lambrecht/Pfalz, Germany). Fast capacitance was fully canceled, and cell capacitance was partially canceled as much as possible by the circuits of the amplifier. Seventy percent of the series resistance of the recording electrode was compensated. High-voltage-activated Ca^2+^ currents of RGCs were induced by a series of 150 ms depolarizing pulses from −40 mV (the holding potential) up to +10 mV in increments of 10 mV, and the average current amplitudes of the voltage pulses during 135–140 ms were detected and analyzed. Data were acquired at a sample rate of 20 kHz, filtered at 2 kHz, and then stored for further analysis. A fast solution exchanger (RSC-160, Bio-Logic, Claix, France) based on a stepping motor, was used for solution transport, with a solution exchange time being ∼5 ms. All experiments were performed at room temperature (20–25°C).

#### MEA recording

The procedures for MEA refer to our previous work,^11^^,^^85^ with minor modifications. Briefly, rats were dark-adapted overnight (> 12 h) and anesthetized by isoflurane under dim red light. Retinas were dissected free from the eyecup and one quarter of the retina was placed photoreceptor-side down on a piece of Anodisc filter membrane (Whatman, Piscataway, NJ, USA). The mounted retina was transferred into the recording chamber of an MEA chip (60MEA200/30iR-ITO-gr, Multi-Channel Systems GmbH, Reutilingen, Germany) and the GCL faced the array. The retina was continuously superfused with oxygenated bicarbonate-buffered Ames’ medium (5–6 mL/min) and maintained at 30 ± 2°C using a temperature controller (TC-324B, Warner Instruments, Hamden, CT, USA). The retina sat in the chamber for 40 min in the dark before beginning recordings to permit stabilization of spike amplitudes. Voltage data were digitized at 10 kHz, amplified and acquired using the USB MEA60 Inv BC System and MC Rack software (Multi Channel Systems). Signals were high-pass filtered at 200 Hz and stored on a personal computer. Retinas were full-field stimulated by 1-s 500-nm light flashes generated using a custom-modified fiber optic LED illuminator (Model 66991, DiCon Fibersoptics Inc., Richmond, CA, USA) with stimulus timing controlled by a logic circuit integrated in the illuminator. The intensity of light stimulation ranged from 3.42 × 10^6^ to 5.86 × 10^10^ photons/cm^2^/s by introducing different neutral density filters (Edmund Optics Inc., Barrington, NJ, USA) into the light path. Inter stimulus intervals increased progressively within the series, ranging from 1 min between the first three dim stimuli to 5 min between the last two brightest ones. The recording of each stimulus was kept for 3 s, with 1 s before, during and after light stimulation.

Spike sorting of the raw recording data followed a protocol previously described^85^ using Offline Sorter software (Plexon Inc., Dallas, TX, USA). The spike detection threshold was set for each channel at 3–4 times the standard deviation of the voltage. The detected spike waveforms were subjected to cluster analysis using the first three principal components, and the resulting clusters were manually corrected for clustering errors. RGCs were classified into three types by their spiking pattern to light stimuli, which was defined by the response dominant index (RDI) according to previous studies.^86^^,^^87^ RDI is computed using the equation: RDI = (R_ON_−R_OFF_)/(R_ON_+R_OFF_), where R_ON_ and R_OFF_ are the spike number during the 1-s light stimulus and 1 s after the light offset respectively, minus the spike number of 1 s preceding the light onset. Cells with an RDI smaller than −0.6 or larger than 0.6 at more than half of light intensities were defined as OFF- and ON-RGCs, respectively; cells with an RDI between −0.6 and 0.6 were defined as ON-OFF RGCs. The total spike number that is R_ON_ (for ON RGC) or R_OFF_ (for OFF RGC) in the equation was analyzed.

#### Chemicals

Ex-4, nimodipine, Ex-9-39 and BayK-8644 were purchased from MedChemExpress (Monmouth Junction, NJ, USA), while TTX, Ryanodine, W-7 and U-73122 were from Tocris Bioscience (Ellisville, MO, USA). NF-449 was purchased from Cayman Chemical (Ann Arbor, MI, USA) and C2 Ceramide from Santa Cruz (Dallas, TX, USA). The others were purchased from Sigma-Aldrich. Nimodipine, Bis-IV, U-73122, ryanodine, W-7, FK-506, OA, and C2 Ceramide were initially dissolved in DMSO for stock. The final concentration of DMSO was less than 0.1%, with no effects on the calcium currents of RGCs. All other drug solutions were prepared in ion-free water, stored at −20°C and freshly diluted to the final concentrations using extracellular or intracellular solutions. For topical ocular administration, Ex-4 and Ex-9-39 were dissolved in saline, and BayK-8644 was dissolved in 50% PEG-400 diluted with saline.

### Quantification and statistical analysis

All data were analyzed using Excel (Microsoft, Redmond, WA, USA), Pulsefit 8.62 (HEKA Elektronik), and GraphPad prism 8 (GraphPad Software, San Diego, CA, USA). To identify significant differences, paired *t* test, unpaired *t* test, one-way ANOVA followed by post hoc Tukey’s multiple comparisons tests and two-way repeated measures (RM) ANOVA followed by post hoc Sidak’s multiple comparisons tests were used. Unless otherwise specified, p values represented the results of the paired or unpaired *t* test. p < 0.05 was considered statistically significant.

## Data Availability

• All Data reported in this paper will be shared by the lead contact upon request. • This paper does not report original code. • Any additional information required to reanalyze the data reported in this paper is available from the lead contact upon request.

## References

[1] Leasher J.L., Bourne R.R.A., Flaxman S.R., Jonas J.B., Keeffe J., Naidoo K., Pesudovs K., Price H., White R.A., Wong T.Y. (2016). Global Estimates on the Number of People Blind or Visually Impaired by Diabetic Retinopathy: A Meta-analysis From 1990 to 2010. Diabetes Care.

[2] Teo Z.L., Tham Y.C., Yu M., Chee M.L., Rim T.H., Cheung N., Bikbov M.M., Wang Y.X., Tang Y., Lu Y. (2021). Global Prevalence of Diabetic Retinopathy and Projection of Burden through 2045: Systematic Review and Meta-analysis. Ophthalmology.

[3] Barber A.J., Baccouche B. (2017). Neurodegeneration in diabetic retinopathy: Potential for novel therapies. Vis. Res..

[4] Gastinger M.J., Kunselman A.R., Conboy E.E., Bronson S.K., Barber A.J. (2008). Dendrite remodeling and other abnormalities in the retinal ganglion cells of Ins2 Akita diabetic mice. Invest. Ophthalmol. Vis. Sci..

[5] Kern T.S., Barber A.J. (2008). Retinal ganglion cells in diabetes. J. Physiol..

[6] Martin P.M., Roon P., Van Ells T.K., Ganapathy V., Smith S.B. (2004). Death of retinal neurons in streptozotocin-induced diabetic mice. Invest. Ophthalmol. Vis. Sci..

[7] Kohzaki K., Vingrys A.J., Bui B.V. (2008). Early inner retinal dysfunction in streptozotocin-induced diabetic rats. Invest. Ophthalmol. Vis. Sci..

[8] Lee R., Wong T.Y., Sabanayagam C. (2015). Epidemiology of diabetic retinopathy, diabetic macular edema and related vision loss. Eye Vis..

[9] Simó R., Stitt A.W., Gardner T.W. (2018). Neurodegeneration in diabetic retinopathy: does it really matter?. Diabetologia.

[10] Bloodworth J.M. (1962). Diabetic retinopathy. Diabetes.

[11] Chen W.Y., Han X., Cui L.J., Yu C.X., Sheng W.L., Yu J., Yuan F., Zhong Y.M., Yang X.L., Weng S.J. (2021). Cell-Subtype-Specific Remodeling of Intrinsically Photosensitive Retinal Ganglion Cells in Streptozotocin-Induced Diabetic Mice. Diabetes.

[12] Sampedro J., Bogdanov P., Ramos H., Solà-Adell C., Turch M., Valeri M., Simó-Servat O., Lagunas C., Simó R., Hernández C. (2019). New Insights into the Mechanisms of Action of Topical Administration of GLP-1 in an Experimental Model of Diabetic Retinopathy. J. Clin. Med..

[13] Amato R., Catalani E., Dal Monte M., Cammalleri M., Cervia D., Casini G. (2022). Morpho-functional analysis of the early changes induced in retinal ganglion cells by the onset of diabetic retinopathy: The effects of a neuroprotective strategy. Pharmacol. Res..

[14] Cui R.Z., Wang L., Qiao S.N., Wang Y.C., Wang X., Yuan F., Weng S.J., Yang X.L., Zhong Y.M. (2019). ON-Type Retinal Ganglion Cells are Preferentially Affected in STZ-Induced Diabetic Mice. Invest. Ophthalmol. Vis. Sci..

[15] Meyer-Rüsenberg B., Pavlidis M., Stupp T., Thanos S. (2007). Pathological changes in human retinal ganglion cells associated with diabetic and hypertensive retinopathy. Graefes Arch. Clin. Exp. Ophthalmol..

[16] Qin Y., Xu G., Wang W. (2006). Dendritic abnormalities in retinal ganglion cells of three-month diabetic rats. Curr. Eye Res..

[17] Furukawa K., Fu W., Li Y., Witke W., Kwiatkowski D.J., Mattson M.P. (1997). The actin-severing protein gelsolin modulates calcium channel and NMDA receptor activities and vulnerability to excitotoxicity in hippocampal neurons. J. Neurosci..

[18] Mattson M.P., Barger S.W., Cheng B., Lieberburg I., Smith-Swintosky V.L., Rydel R.E. (1993). beta-Amyloid precursor protein metabolites and loss of neuronal Ca2+ homeostasis in Alzheimer's disease. Trends Neurosci..

[19] Wasterlain C.G., Fujikawa D.G., Penix L., Sankar R. (1993). Pathophysiological mechanisms of brain damage from status epilepticus. Epilepsia.

[20] Araie M., Mayama C. (2011). Use of calcium channel blockers for glaucoma. Prog. Retin. Eye Res..

[21] Crish S.D., Calkins D.J. (2011). Neurodegeneration in glaucoma: progression and calcium-dependent intracellular mechanisms. Neuroscience.

[22] Vallazza-Deschamps G., Fuchs C., Cia D., Tessier L.H., Sahel J.A.A., Dreyfus H., Picaud S. (2005). Diltiazem-induced neuroprotection in glutamate excitotoxicity and ischemic insult of retinal neurons. Doc. Ophthalmol..

[23] Uemura A., Mizota A. (2008). Retinal concentration and protective effect against retinal ischemia of nilvadipine in rats. Eur. J. Ophthalmol..

[24] Sargoy A., Sun X., Barnes S., Brecha N.C. (2014). Differential calcium signaling mediated by voltage-gated calcium channels in rat retinal ganglion cells and their unmyelinated axons. PLoS One.

[25] Karschin A., Lipton S.A. (1989). Calcium channels in solitary retinal ganglion cells from post-natal rat. J. Physiol..

[26] Holst J.J. (2007). The physiology of glucagon-like peptide 1. Physiol. Rev..

[27] Koshal P., Jamwal S., Kumar P. (2018). Glucagon-like Peptide-1 (GLP-1) and neurotransmitters signaling in epilepsy: An insight review. Neuropharmacology.

[28] Salcedo I., Tweedie D., Li Y., Greig N.H. (2012). Neuroprotective and neurotrophic actions of glucagon-like peptide-1: an emerging opportunity to treat neurodegenerative and cerebrovascular disorders. Br. J. Pharmacol..

[29] Hernández C., Bogdanov P., Corraliza L., García-Ramírez M., Solà-Adell C., Arranz J.A., Arroba A.I., Valverde A.M., Simó R. (2016). Topical Administration of GLP-1 Receptor Agonists Prevents Retinal Neurodegeneration in Experimental Diabetes. Diabetes.

[30] Shu X., Zhang Y., Li M., Huang X., Yang Y., Zeng J., Zhao Y., Wang X., Zhang W., Ying Y. (2019). Topical ocular administration of the GLP-1 receptor agonist liraglutide arrests hyperphosphorylated tau-triggered diabetic retinal neurodegeneration via activation of GLP-1R/Akt/GSK3β signaling. Neuropharmacology.

[31] Fischer A.J., Stanke J.J., Ghai K., Scott M., Omar G. (2007). Development of bullwhip neurons in the embryonic chicken retina. J. Comp. Neurol..

[32] Cai X., Li J., Wang M., She M., Tang Y., Li J., Li H., Hui H. (2017). GLP-1 Treatment Improves Diabetic Retinopathy by Alleviating Autophagy through GLP-1R-ERK1/2-HDAC6 Signaling Pathway. Int. J. Med. Sci..

[33] Fan Y., Liu K., Wang Q., Ruan Y., Zhang Y., Ye W. (2014). Exendin-4 protects retinal cells from early diabetes in Goto-Kakizaki rats by increasing the Bcl-2/Bax and Bcl-xL/Bax ratios and reducing reactive gliosis. Mol. Vis..

[34] Simó R., Hernández C. (2017). GLP-1R as a Target for the Treatment of Diabetic Retinopathy: Friend or Foe?. Diabetes.

[35] Zhang Y., Wang Q., Zhang J., Lei X., Xu G.T., Ye W. (2009). Protection of exendin-4 analogue in early experimental diabetic retinopathy. Graefes Arch. Clin. Exp. Ophthalmol..

[36] Zhang Y., Zhang J., Wang Q., Lei X., Chu Q., Xu G.T., Ye W. (2011). Intravitreal injection of exendin-4 analogue protects retinal cells in early diabetic rats. Invest. Ophthalmol. Vis. Sci..

[37] Gilman C.P., Perry T., Furukawa K., Grieg N.H., Egan J.M., Mattson M.P. (2003). Glucagon-like peptide 1 modulates calcium responses to glutamate and membrane depolarization in hippocampal neurons. J. Neurochem..

[38] Hölscher C. (2012). Potential role of glucagon-like peptide-1 (GLP-1) in neuroprotection. CNS Drugs.

[39] Hunter K., Hölscher C. (2012). Drugs developed to treat diabetes, liraglutide and lixisenatide, cross the blood brain barrier and enhance neurogenesis. BMC Neurosci..

[40] Kastin A.J., Akerstrom V. (2003). Entry of exendin-4 into brain is rapid but may be limited at high doses. Int. J. Obes. Relat. Metab. Disord..

[41] Kastin A.J., Akerstrom V., Pan W. (2002). Interactions of glucagon-like peptide-1 (GLP-1) with the blood-brain barrier. J. Mol. Neurosci..

[42] McClean P.L., Parthsarathy V., Faivre E., Hölscher C. (2011). The diabetes drug liraglutide prevents degenerative processes in a mouse model of Alzheimer's disease. J. Neurosci..

[43] McGovern S.F.J., Hunter K., Hölscher C. (2012). Effects of the glucagon-like polypeptide-1 analogue (Val8)GLP-1 on learning, progenitor cell proliferation and neurogenesis in the C57B/16 mouse brain. Brain Res..

[44] Mentlein R. (1999). Dipeptidyl-peptidase IV (CD26)--role in the inactivation of regulatory peptides. Regul. Pept..

[45] Nadal-Nicolás F.M., Jiménez-López M., Sobrado-Calvo P., Nieto-López L., Cánovas-Martínez I., Salinas-Navarro M., Vidal-Sanz M., Agudo M. (2009). Brn3a as a marker of retinal ganglion cells: qualitative and quantitative time course studies in naive and optic nerve-injured retinas. Invest. Ophthalmol. Vis. Sci..

[46] Doyle M.E., Egan J.M. (2007). Mechanisms of action of glucagon-like peptide 1 in the pancreas. Pharmacol. Ther..

[47] Hölscher C. (2010). The role of GLP-1 in neuronal activity and neurodegeneration. Vitam. Horm..

[48] Zhang T., Ruan H.Z., Wang Y.C., Shao Y.Q., Zhou W., Weng S.J., Zhong Y.M. (2022). Signaling Mechanism for Modulation by GLP-1 and Exendin-4 of GABA Receptors on Rat Retinal Ganglion Cells. Neurosci. Bull..

[49] Chik C.L., Liu Q.Y., Li B., Klein D.C., Zylka M., Kim D.S., Chin H., Karpinski E., Ho A.K. (1997). Alpha 1D L-type Ca(2+)-channel currents: inhibition by a beta-adrenergic agonist and pituitary adenylate cyclase-activating polypeptide (PACAP) in rat pinealocytes. J. Neurochem..

[50] Stella S.L., Thoreson W.B. (2000). Differential modulation of rod and cone calcium currents in tiger salamander retina by D2 dopamine receptors and cAMP. Eur. J. Neurosci..

[51] Armstrong D.L. (1989). Calcium channel regulation by calcineurin, a Ca2+-activated phosphatase in mammalian brain. Trends Neurosci..

[52] Shigeto M., Cha C.Y., Rorsman P., Kaku K. (2017). A role of PLC/PKC-dependent pathway in GLP-1-stimulated insulin secretion. J. Mol. Med..

[53] Voitenko N.V., Kruglikov I.A., Kostyuk E.P., Kostyuk P.G. (2000). Effect of streptozotocin-induced diabetes on the activity of calcium channels in rat dorsal horn neurons. Neuroscience.

[54] Acuna-Goycolea C., van den Pol A. (2004). Glucagon-like peptide 1 excites hypocretin/orexin neurons by direct and indirect mechanisms: implications for viscera-mediated arousal. J. Neurosci..

[55] Xiao Y.F., Nikolskaya A., Jaye D.A., Sigg D.C. (2011). Glucagon-like peptide-1 enhances cardiac L-type Ca2+ currents via activation of the cAMP-dependent protein kinase A pathway. Cardiovasc. Diabetol..

[56] Kusari J., Zhou S., Padillo E., Clarke K.G., Gil D.W. (2007). Effect of memantine on neuroretinal function and retinal vascular changes of streptozotocin-induced diabetic rats. Invest. Ophthalmol. Vis. Sci..

[57] Vilsbøll T., Agersø H., Krarup T., Holst J.J. (2003). Similar elimination rates of glucagon-like peptide-1 in obese type 2 diabetic patients and healthy subjects. J. Clin. Endocrinol. Metab..

[58] Göke R., Fehmann H.C., Linn T., Schmidt H., Krause M., Eng J., Göke B. (1993). Exendin-4 is a high potency agonist and truncated exendin-(9-39)-amide an antagonist at the glucagon-like peptide 1-(7-36)-amide receptor of insulin-secreting beta-cells. J. Biol. Chem..

[59] Thorens B., Porret A., Bühler L., Deng S.P., Morel P., Widmann C. (1993). Cloning and functional expression of the human islet GLP-1 receptor. Demonstration that exendin-4 is an agonist and exendin-(9-39) an antagonist of the receptor. Diabetes.

[60] Yamamoto H., Kishi T., Lee C.E., Choi B.J., Fang H., Hollenberg A.N., Drucker D.J., Elmquist J.K. (2003). Glucagon-like peptide-1-responsive catecholamine neurons in the area postrema link peripheral glucagon-like peptide-1 with central autonomic control sites. J. Neurosci..

[61] Yamamoto H., Lee C.E., Marcus J.N., Williams T.D., Overton J.M., Lopez M.E., Hollenberg A.N., Baggio L., Saper C.B., Drucker D.J., Elmquist J.K. (2002). Glucagon-like peptide-1 receptor stimulation increases blood pressure and heart rate and activates autonomic regulatory neurons. J. Clin. Invest..

[62] Ramos H., Bogdanov P., Sampedro J., Huerta J., Simó R., Hernández C. (2020). Beneficial Effects of Glucagon-Like Peptide-1 (GLP-1) in Diabetes-Induced Retinal Abnormalities: Involvement of Oxidative Stress. Antioxidants.

[63] Nelson R., Kolb H. (2004). ON and OFF pathways in the vertebrate retina and visual system. Vis. Neurosci..

[64] Aung M.H., Park H.N., Han M.K., Obertone T.S., Abey J., Aseem F., Thule P.M., Iuvone P.M., Pardue M.T. (2014). Dopamine deficiency contributes to early visual dysfunction in a rodent model of type 1 diabetes. J. Neurosci..

[65] Della Sala S., Bertoni G., Somazzi L., Stubbe F., Wilkins A.J. (1985). Impaired contrast sensitivity in diabetic patients with and without retinopathy: a new technique for rapid assessment. Br. J. Ophthalmol..

[66] Umino Y., Solessio E. (2013). Loss of scotopic contrast sensitivity in the optomotor response of diabetic mice. Invest. Ophthalmol. Vis. Sci..

[67] Dauner D.G., Farley J.F. (2021). Comparing the use of individual and composite terms to evaluate adverse drug event disproportionality: a focus on glucagon-like peptide-1 receptor agonists and diabetic retinopathy. Expet Opin. Drug Saf..

[68] Marso S.P., Bain S.C., Consoli A., Eliaschewitz F.G., Jódar E., Leiter L.A., Lingvay I., Rosenstock J., Seufert J., Warren M.L. (2016). Semaglutide and Cardiovascular Outcomes in Patients with Type 2 Diabetes. N. Engl. J. Med..

[69] Smits M.M., Van Raalte D.H. (2021). Safety of Semaglutide. Front. Endocrinol..

[70] Bethel M.A., Diaz R., Castellana N., Bhattacharya I., Gerstein H.C., Lakshmanan M.C. (2021). HbA(1c) Change and Diabetic Retinopathy During GLP-1 Receptor Agonist Cardiovascular Outcome Trials: A Meta-analysis and Meta-regression. Diabetes Care.

[71] Vilsbøll T., Bain S.C., Leiter L.A., Lingvay I., Matthews D., Simó R., Helmark I.C., Wijayasinghe N., Larsen M. (2018). Semaglutide, reduction in glycated haemoglobin and the risk of diabetic retinopathy. Diabetes Obes. Metabol..

[72] Simó R., Franch-Nadal J., Vlacho B., Real J., Amado E., Flores J., Mata-Cases M., Ortega E., Rigla M., Vallés J.A. (2023). Rapid Reduction of HbA1c and Early Worsening of Diabetic Retinopathy: A Real-World Population-Based Study in Subjects With Type 2 Diabetes. Diabetes Care.

[73] Simó R., Hernández C. (2023). What else can we due to prevent diabetic retinopathy?. Diabetologia.

[74] Aizu Y., Oyanagi K., Hu J., Nakagawa H. (2002). Degeneration of retinal neuronal processes and pigment epithelium in the early stage of the streptozotocin-diabetic rats. Neuropathology.

[75] Wang L., Deng Q.Q., Wu X.H., Yu J., Yang X.L., Zhong Y.M. (2013). Upregulation of glutamate-aspartate transporter by glial cell line-derived neurotrophic factor ameliorates cell apoptosis in neural retina in streptozotocin-induced diabetic rats. CNS Neurosci. Ther..

[76] Ramzy A.R., Nausheen S., Chelikani P.K. (2014). Ileal transposition surgery produces ileal length-dependent changes in food intake, body weight, gut hormones and glucose metabolism in rats. Int. J. Obes..

[77] Kanwar M., Kowluru R.A. (2009). Role of glyceraldehyde 3-phosphate dehydrogenase in the development and progression of diabetic retinopathy. Diabetes.

[78] Marques T.E.B.S., de Mendonça L.R., Pereira M.G., de Andrade T.G., Garcia-Cairasco N., Paçó-Larson M.L., Gitaí D.L.G. (2013). Validation of suitable reference genes for expression studies in different pilocarpine-induced models of mesial temporal lobe epilepsy. PLoS One.

[79] Chen S.N., Xu Z.G., Ma Y.X., Chen S., He G.H., Han M., Gao X., Wang J.H., Wu B., Wang J. (2021). Protective effect of LIF-huMSCs on the retina of diabetic model rats. Int. J. Ophthalmol..

[80] Livak K.J., Schmittgen T.D. (2001). Analysis of relative gene expression data using real-time quantitative PCR and the 2(-Delta Delta C(T)) Method. Methods.

[81] He Y.Y., Wang L., Zhang T., Weng S.J., Lu J., Zhong Y.M. (2020). Aerobic exercise delays retinal ganglion cell death after optic nerve injury. Exp. Eye Res..

[82] Deng Q.Q., Sheng W.L., Zhang G., Weng S.J., Yang X.L., Zhong Y.M. (2016). Signalling mechanism for somatostatin receptor 5-mediated suppression of AMPA responses in rat retinal ganglion cells. Neuropharmacology.

[83] Liu F., Weng S.J., Yang X.L., Zhong Y.M. (2015). Orexin-A potentiates L-type calcium/barium currents in rat retinal ganglion cells. Neuroscience.

[84] Hagiwara S., Ohmori H. (1982). Studies of calcium channels in rat clonal pituitary cells with patch electrode voltage clamp. J. Physiol..

[85] Weng S., Wong K.Y., Berson D.M. (2009). Circadian modulation of melanopsin-driven light response in rat ganglion-cell photoreceptors. J. Biol. Rhythm..

[86] Liu F., Zhang J., Xiang Z., Xu D., So K.F., Vardi N., Xu Y. (2018). Lycium Barbarum Polysaccharides Protect Retina in rd1 Mice During Photoreceptor Degeneration. Invest. Ophthalmol. Vis. Sci..

[87] Tian N., Copenhagen D.R. (2003). Visual stimulation is required for refinement of ON and OFF pathways in postnatal retina. Neuron.

