# Supplementary material for: Exendin-4 promotes retinal ganglion cell survival and function by inhibiting calcium channels in experimental diabetes
Source: iScience. 2023 Aug 18;26(9):107680. doi: 10.1016/j.isci.2023.107680 (PMC10481356; doi:10.1016/j.isci.2023.107680)
Supplement: Document S1. Figures S1–S4 [file mmc1.pdf]

iScience, Volume *26*

Supplemental information

Exendin-4 promotes retinal ganglion cell survival and function by inhibiting calcium channels in experimental diabetes

Yong-Chen Wang, Lu Wang, Yu-Qi Shao, Shi-Jun Weng, Xiong-Li Yang, and Yong-Mei Zhong

Supplemental Information

Supplemental Figures

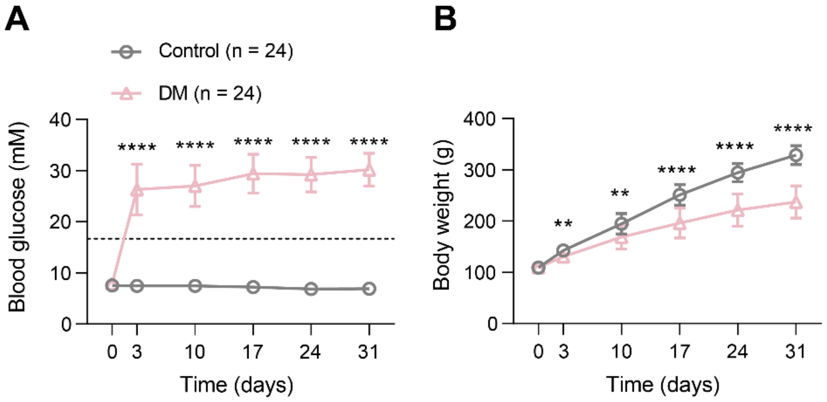

Figure S1. Blood glucose levels and body weights of control and DM rats, related to STAR Methods. (**A**) Control and DM rats had similar blood glucose levels before injection of STZ (day 0). Control rats (n = 24) maintained blood glucose levels at ~7 mM over the course of the study, whereas blood glucose levels in the STZ-treated rats (n = 24) were increased by more than threefold at all five time points. The black dashed line indicates a blood glucose level of 16.7 mM. (**B**) Control rats gained weight over the course of 4 weeks, whereas DM rats gained less weight (**$p < 0.01$ and ****$p < 0.0001$ vs. control by Sidak's multiple comparisons test after two-way RM ANOVA). Data are presented as mean ± SD.

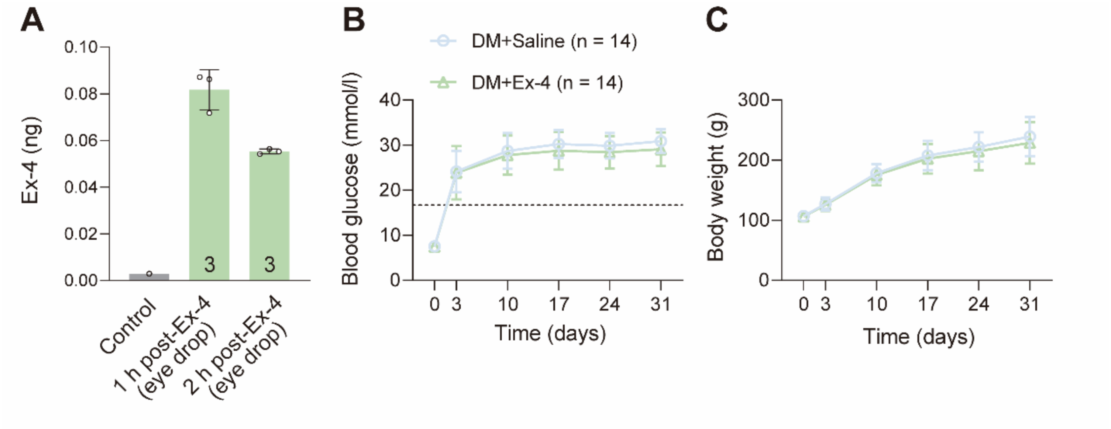

Figure S2. Topical administration of Ex-4 by eye drops could reach the neural retina and did not change the blood glucose levels and body weights of DM rats, related to Figure 2. (**A**) ELISA showing the increased Ex-4 levels in the retinas 1 (n = 3) and 2 hours (n = 3) after Ex-4 administration by eye drops. (**B**) DM rats treated with Ex-4 (n = 14) maintained high levels of blood glucose similar to those of DM rats treated with saline (n = 14) over the course of the study ($p > 0.05$ by two-way RM ANOVA). (**C**) The body weights of DM+Saline rats and DM+Ex-4 rats were similar over the course of the study ($p > 0.05$). Data are presented as mean ± SD.

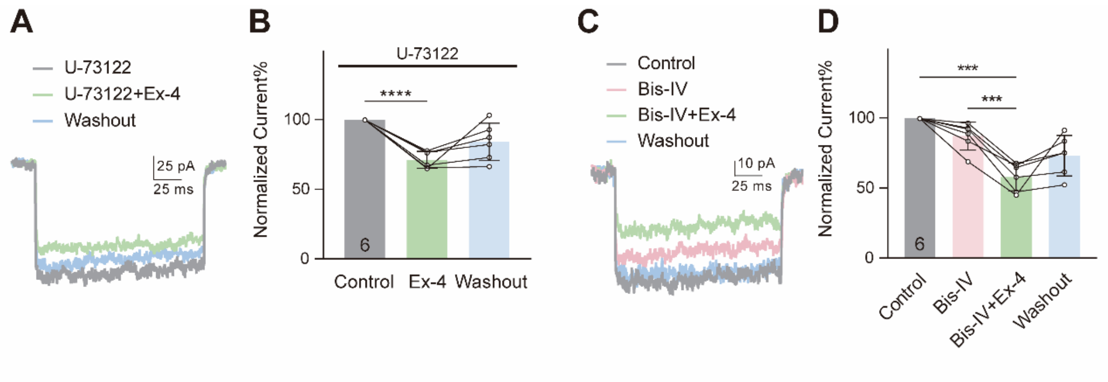

Figure S3. Ex-4-induced suppression of I_{LCa} is independent of the PLC-PKC signaling pathway, related to Figure 6. (**A, C**) Current traces of two RGCs, showing that Ex-4 still suppressed the I_{LCa} during internal dialysis of 10 μM U-73122 (**A**) or during perfusion of Bis-IV (10 μM) (**C**), and the corresponding summary data are shown in (**B)** (n = 6) and (**D)** (n = 6). Data are presented as mean ± SD, n.s., p > 0.05, ***p < 0.001 and ****p < 0.0001 by paired *t* test.

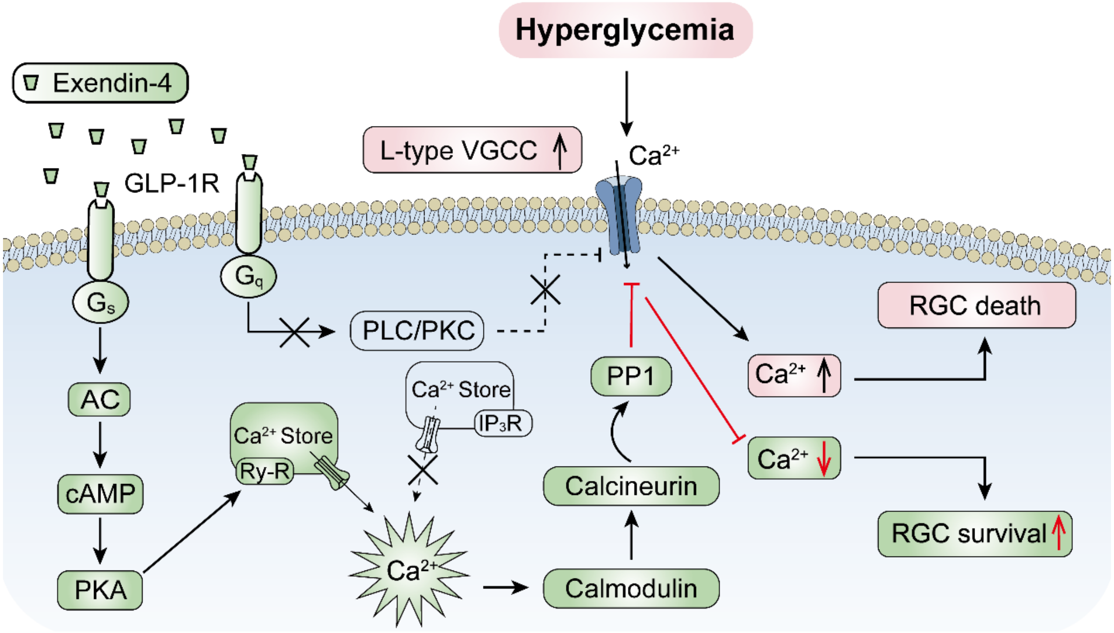

Figure S4. Schematic diagram illustrating the putative signaling pathway that Ex-4 can rescue RGCs at the early stage of diabetes by regulating L-VGCCs, related to Figures 1–6. Hyperglycemia results in the increase of I_{LCa} density and Ca^{2+} influx in RGCs, thus inducing the death of RGCs. By activating Gs protein-coupled GLP-1R, Ex-4 suppresses I_{LCa} via a distinct intracellular cAMP-PKA/ryanodine/Ca^{2+}/CaM/calcineurin/PP1 signaling pathway, which could reduce Ca^{2+} influx and promote diabetic RGC survival. The PLC-PKC signaling pathway is not involved in the Ex-4 effect.
